# Acyl-CoA thioesterase 1 prevents cardiomyocytes from Doxorubicin-induced ferroptosis via shaping the lipid composition

**DOI:** 10.1038/s41419-020-02948-2

**Published:** 2020-09-15

**Authors:** Yunchang Liu, Liping Zeng, Yong Yang, Chen Chen, Daowen Wang, Hong Wang

**Affiliations:** 1 Division of Cardiology, Department of Internal Medicine, Tongji Hospital, Tongji Medical College, Huazhong University of Science and Technology, 430030 Wuhan, China; 2 Hubei Key Laboratory of Genetics and Molecular Mechanisms of Cardiological Disorders, 430030 Wuhan, China; 3 Department of Biochemistry and Molecular Biology, Tongji Medical College, Huazhong University of Science and Technology, 430030 Wuhan, China

**Keywords:** Cell death, Fatty acids, Transcriptomics

## Abstract

In this study, we first established the doxorubicin-induced cardiotoxicity (DIC) model with C57BL/6 mice and confirmed cardiac dysfunction with transthoracic echocardiography examination. RNA-sequencing was then performed to explore the potential mechanisms and transcriptional changes in the process. The metabolic pathway, biosynthesis of polyunsaturated fatty acid was significantly altered in DOX-treated murine heart, and Acot1 was one of the leading-edge core genes. We then investigated the role of Acot1 to ferroptosis that was reported recently to be related to DIC. The induction of ferroptosis in the DOX-treated heart was confirmed by transmission electron microscopy, and the inhibition of ferroptosis using Fer-1 effectively prevented the cardiac injury as well as the ultrastructure changes of cardiomyocyte mitochondrial. Both in vitro and in vivo experiments proved the downregulation of Acot1 in DIC, which can be partially prevented with Fer-1 treatment. Overexpression of Acot1 in cell lines showed noteworthy protection to ferroptosis, while the knock-down of Acot1 sensitized cardiomyocytes to ferroptosis by DIC. Finally, the heart tissue of αMHC-Acot1 transgenic mice presented altered free fatty acid composition, indicating that the benefit of Acot1 in the inhibition of ferroptosis lies biochemically and relates to its enzymatic function in lipid metabolism in DIC. The current study highlights the importance of ferroptosis in DIC and points out the potential protective role of Acot1 in the process. The beneficial role of Acot1 may be related to its biochemical function by shaping the lipid composition. In all, Acot1 may become a potential treating target in preventing DIC by anti-ferroptosis.

## Introduction

Doxorubicin (DOX), has been reported to induce various forms of regulated cell death, including apoptosis, necroptosis, and pyroptosis, of terminally differentiated cardiomyocytes, which is a vital factor in the occurrence of myocardial injury^1–3^. Dox-induced cardiotoxicity (DIC) has been ascribed to disrupted iron metabolism (iron overload) and excesses of reactive oxygen species (ROS). Generation of ROS by iron dysregulation, which may damage DNA, protein, lipid composition of membrane structures, and eventually accelerates the death of cardiomyocytes. However, studies have witnessed the failures of most kinds of ‘antioxidants’ as treatments for DIC^4,5^. Emerging studies showed that it was not ROS scavenging but the induction of an unrevealed mitochondrial redox signaling, that casts protective effect in the mito-Q (mitochondria-targeted form of CoQ10) treatment of DIC^6^. In all, patterns of cell death that have not been fully elucidated may also contribute to DIC.

Ferroptosis, a novel form of regulated cell death, is characterized by an iron-dependent increase in lipid peroxidation (with lipid hydroperoxides and aldehydes formation) and damage of biological membrane structures^7,8^. Studies have shown that the oxidation of ω-6 polyunsaturated fatty acid (PUFA) based phosphatidylethanolamines (PEs) is the crucial procedure of the induction of ferroptosis and genes that influence the formation of PUFA-CoA (acyl-CoA synthetase long-chain family member 4, Acsl4) or the insertion of PUFA-CoA to PEs in cells (lysophosphatidylcholine acyltransferase 3, Lpcat3) may influence the induction of ferroptosis^9^. Though most of the researches focuses on the role of ferroptosis in cancer diseases, a recent one has linked cell death in DIC to ferroptosis. It was considered that the up-regulation of Nrf2/Hmox1 in DIC disarranged the homeostasis of iron metabolism and increased the process of ferroptosis in cardiomyocytes^10^. However, few studies have investigated the role of other enzymes of PUFA metabolism-related pathways in the induction of ferroptosis.

Acyl-CoA thioesterase 1 (Acot1), as an important enzyme in fatty acid metabolism, catalyzes the reaction of fatty acyl-CoAs to CoA-SH and free fatty acids^11^. Due to the high energy demand to supply circulating function, cardiomyocytes prefer fatty acids as metabolism substrates and possess massive of mitochondria for oxidation^12^. Mitochondria are considered to be one of the majority sources of lipid peroxidation, which prompt cardiomyocytes to gain a higher capacity for lipid-ROS production^13^. Our researches before have shown that Acot1 can reduce the oxidative stress in cardiomyocytes and protect cardiac function in many disease models, including LPS-induced cardiac injury and diabetic cardiomyopathy^14,15^. Enzymatically, Acot1 catalyze the opposite biochemical process of which is mediated by ACSL4, thus, may perform potential anti-ferroptosis ability. Whether Acot1 contributes to the inhibition of lipid-ROS formation and ferroptosis in doxorubicin cardiotoxicity has not been investigated.

In the current study, we initially performed both in vivo and in vitro cardiac cytotoxicity models of DOX to explore the role of ferroptosis and the possible mechanism in this process. We identified that biosynthesis of polyunsaturated fatty acid was one of the most down-regulated bio-pathways and Acot1 was one of the most down-regulated genes in the DOX-treated murine heart with RNA-seq based bioinformatic analysis. We hypothesized that Acot1 might play a role in DIC by protecting ferroptosis. GC-MS was then used to explore the function of modified Acot1 expression in cardiomyocytes and the molecular mechanism of Acot1 in inhibiting ferroptosis. We found that modified expression of Acot1 caused the reshape of lipid composition in murine heart and subsequently affected the sensitivity of cardiomyocytes to ferroptosis. Evidence in HL-1 cardiomyocytes cell line supported that Acot1 inhibited lipid peroxidation and prevented cells from ferroptosis in DIC. Our results provided insights into the novel pathogenic mechanism of ferroptosis in the process of DIC and possible therapeutic target for the clinical practice.

## Materials and methods

### Chemicals and reagents

Chemicals and reagents were from MedChemExpress including: Doxorubicin (#HY-15142A, DOX), Ferrostatin-1 (#HY-100579, Fer-1), (1S,3R)-RSL3 (#HY-100218A, RSL-3), Stearic acid (#HY-B2219), and Arachidonic acid (#HY-109590), Docosahexaenoic Acid (#HY-B2167). Acot1 siRNAs and negative control were from RiboBio (China). DNA ladders, pre-stained protein markers were from Thermo Fisher Scientific (USA).

### C57BL/6 mice

All animal experimental protocols were approved by the Institutional Animal Research Committee of Tongji Medical College and following the ARRIVE Guidelines. C57BL/6 male mice (20–25 g) at 7 weeks were purchased from the Vital River Laboratory Animal Technology Co., Ltd. Mice were housed under specific pathogen-free conditions. After adaptively feeding for 1 week, all the mice were divided into different groups randomly and the experiment was then conducted. No blinding was done in animal experiments.

### Doxorubicin-induced sub-acute cardiac injury model

A modified protocol was used to establish an in vivo sub-acute DIC model with a 25-mg/kg cumulative dose. Briefly, two doses of DOX was administrated by intraperitoneal injection, 15 mg/kg at Day 1, and 10 mg/kg at Day 8. Mice were then killed at Day 15 after transthoracic echocardiography examination. Murine organs were then collected, frozen in liquid nitrogen followed by storage at −80 °C for further experiments or fixed with 4% paraformaldehyde for the histological analysis.

### Echocardiography

After anesthesia at Day 15, the myocardial function of different groups was assessed using Vevo770® high-resolution imaging system with a 30-MHz high-frequency scan-head (Visual Sonics, Canada) as previously described^16^. And data were then analyzed with its supporting software packages.

### RNA-Seq analysis

RNA samples of murine heart tissue were collected from C57BL/6 mice treated with or without DOX and stored with RNAStore solution at 4 °C (#DP408-02, Tiangen Biotech Co., Ltd.). RNA isolation, quality control, library construction, and sequencing were performed by the Beijing Genomics Institute (www.genomics.org.cn, BGI) using the BGISEQ-500 platform. Cleaned reads were then mapped to the GRCm38.P6 reference genome with Bowtie2 (v2.2.5)^17^. Transcript abundances were measured with RSEM (v1.2.8)^18^. Further bioinformatics analyses were all accomplished in RStudio with R (v3.5.3) and GSEA (v4.0.3) with GSKB (v1.14.0)^19–21^. Low count genes were removed by a cut-off at 0.5829 FPKM. Differential expression analyses were performed with limma (R packages v3.38.3) by a cut-off at 1.44-fold-change and *P* value < 0.05^22^. KEGG pathway enrichment was performed with clusterProfiler (v3.10.1)^23^. Plots performed with ggplot2 (v3.2.1) and ggpubr (v0.2.4)^24,25^. Heatmap of 50 up-regulated genes and 50 down-regulated genes was plotted with pheatmap (v1.0.12)^26^. All codes are available from the corresponding author upon request.

### Histology staining

Murine heart tissue samples were firstly fixed with 4% paraformaldehyde and embedded into paraffin. Tissue sections were then subjected to 4 mm thick for next Hematoxylin-Eosin (HE) and Sirius Red staining. Cross area of cardiomyocytes and collagen area percentages were calculated using Fiji-ImageJ (v1.52p)^27^.

### Transmission electron microscopy

Murine heart tissue samples were fixed with a 2.5% glutaraldehyde solution for 6 h at 4 °C. Samples were washed three times in cold 0.1 M phosphate buffer (PH 7.4) for 15 min, then fixed for 2 h with 0.1 M phosphate acid buffer (PH 7.4) containing 1% osmic acid at room temperature, and again washed three times in cold 0.1 M phosphate buffer (PH 7.4) for 15 min. Then samples were dehydrated with a gradient ethanol solution, infiltrated with acetone-epoxy resin, and finally embedded in epoxy resin. The samples were polymerized in a 60 °C oven for 48 h. Embedded samples were cut into ultrathin sections (100 nm thickness) using a Leica EM UC7 microtome. The specimens then underwent uranium-lead (3% uranyl acetate for 15 min and lead lemon solution for 10 min) double-staining at room temperature and dried overnight. Slides were examined with a Tecnai G^2^ 20 TWIN transmission electron microscope (FEI, USA) at an accelerating voltage of 200 kV. Digital images were obtained using its supporting software packages.

### MDA concentration

The concentration of malondialdehyde (MDA) in murine heart tissues was detected using the corresponding kits (#A003-1-2, Nanjing Jiancheng Bioengineering Institute, China), according to the instructions. MDA concentration was normalized to protein concentrations, which were detected by the BCA method with the kit (#AR0197, Boster Biological &Technology Co., Ltd., China).

### HL-1 cell culture, transfection, and treatment

HL-1 cells were cultured in supplemented Claycomb Medium (#51800C, Sigma-Aldrich, USA). Before most experiments, 70–80% confluence was reached. In Acot1 knock-down experiments, HL-1 cells were plated into 12-well plate and transfected with siRNAs against mice Acot1 (si-Acot1, 100 nM) or negative control (si-NC, 100 nM) independently using Lipofectamine 2000 (#11668030, Thermo Fisher Scientific) in Opti-MEM (#31985088, Thermo Fisher Scientific) according to the manufacturer’s protocol. In Acot1 overexpression experiments, HL-1 cells were plated into 12-well plate and then transfected with pcDNA 3.1(+)-Acot1 plasmids (Acot1-OE, 200 ng/well) or vector of pcDNA 3.1(+) (vector, 200 ng/well) independently using Lipofectamine 2000 in Opti-MEM, according to the manufacturer’s protocol. In fatty acids treatment experiments, different concentrations of Stearic acid, Arachidonic acid, and Docosahexaenoic Acid were incubated with HL-1 cells overnight. The details about the treatment of HL-1 cardiomyocytes were described. In most experiments, HL-1 cardiomyocytes were treated with 2 μM DOX or 5 μM RSL-3 dissolved in DMSO for 24 h or 6 h.

### Cell viability assay

HL-1 cardiomyocytes were seeded in 96-well plates and assays of cell viability were detected after 24 h of treatment by the cell counting kit-8 (CCK-8; #P5090, Wuhan Promoter Biological Co., Ltd., China). After indicated concentrations of DOX treatment, a total of 100 μl serum-free Claycomb Medium containing 10 μl CCK-8 solution was added to each well and incubated at 37 °C for 2 h. Then the absorbance at 450 nm was measured with a microplate reader (BioTek Instruments, USA). Cell viability were all normalized to relative control.

### Intracellular glutathione measurement

The concentration of GS-SG and total glutathione in different samples were measured using the GSH and GSSG Assay Kit (#S0053, Beyotime Biotechnology, China) according to the manufacturer’s protocol.

### Lipid peroxidation assessment using C11-BODIPY 581/591

Flow cytometric: HL-1 cardiomyocytes were seeded in 12-well plates. After DOX treated for 6 h, cells were trypsinized, washed with sterile HBSS for 2 times, and then incubated with 2 μM C11-BODIPY (581/591) in serum-free Claycomb Medium at 37 °C for 30 min. Cells were then pelleted, washed with sterile HBSS for 2 times, resuspended in HBSS, and detected with the flow cytometer (BD Accuri™ C6, Becton Dickinson, USA). A minimum of 10,000 live cells gated with blank control was analyzed per condition. FL-1 channel was considered to present ox-BODIPY signal and data were then analyzed by FlowJo (v10.0.7).

Fluorescent image: HL-1 cardiomyocytes were seeded in Confocal Dishes (Coverglass Bottom Dish, Solarbio life science, China). After DOX treated for 6 h, cells were washed with sterile HBSS two times, and then incubated with 2 μM C11-BODIPY (581/591) in serum-free Claycomb Medium at 37 °C for 30 min. Dishes were then washed and fluorescent images were acquired with the confocal microscope (LSM780, ZEISS, USA).

### Western blotting

The protocol of western blotting analysis was as previously described^14^. After treated HL-1 cells were harvested and protein was extracted, protein concentrations from different samples were determined with the BCA protein assay kit. Samples were fractionated by 10% SDS-PAGE and transferred to activated Polyvinylidene difluoride (PVDF) membranes (Bio-Rad Laboratories, USA). Membranes were blocked with 5% FBS in TBST (Tris-buffered saline solution with 0.1% Tween-20) for 2 h at room temperature and then incubated with 1:1000 TBST diluted primary Anti-ACOT1 antibody (#ab100915, Abcam, UK). Images of visualized blot bands were acquired and Gapdh was used as the loading control.

### RealTime-qPCR

The protocol of RealTime-qPCR was as previously described^14^. After total RNA of treated HL-1 cells or cardiac tissues was extracted using RNAiso plus reagent (#9108, TaKaRa, China) following manufacturer’s instructions, cDNA was acquired with PrimeScript™ RT reagent Kit with gDNA Eraser (#RR047A, Takara, China). Later, qPCR was performed using an SYBR Premix Ex Taq II (#RR820A, TaKaRa, China) to detected the targeted mRNAs levels with the 7900HT Fast RealTime PCR system (Applied Biosystems, USA) and Gapdh was used as the internal control. Each reaction was performed in triplicate, and fold change analysis was performed by the 2^−ΔΔCt^ method.

The primer sequences involved in RealTime-qPCR are as follows:

**Table:** 

Gene	Forward (5′→3′)	Reverse (5′→3′)
*Gapdh*	CATCACTGCCACCCAGAAGACTG	ATGCCAGTGAGCTTCCCGTTCAG
*Acot1*	TGATGGTTTGGAGGTTGGGG	TGAAACTCCATTCCCAGCCC
*Ptgs2*	TGGGCCATGGAGTGGACTTA	TCTCAGGGATGTGAGGAGGG

### αMHC-Acot1 transgenic mice

The generation of αMHC-Acot1 transgenic mice on the C57BL/6 background was described previously^14^. Mice 8 weeks of age were killed and heart tissues were then collected, frozen in liquid nitrogen followed by storage at −80 °C for GC-MS detection.

### Total fatty acids extraction

The samples of the murine hearts were mixed with 2 ml of acidified methanol and incubated at 80 °C for 30 min. After methyl esterification, the samples were extracted with 1 ml of hexane and washed with 5 ml of ddH_2_O. Sodium sulfate was used to remove remained water. Next, 500 μl supernatant of the extract was mixed with 25 μl of internal standard (NU-CHEK-PREP methyl esterified fatty acids mixture added with methyl Salicylate) before injection.

### Free fatty acids quantification with GC-MS

Each 1 μl of the extracted sample was analyzed by GC-MS operating in a single ion monitoring mode by Shanghai Applied Protein Technology Co., Ltd. In detail, separation and detection were made by Agilent 7890A/5975C system (Agilent) with Agilent DB-WAX column (Agilent, 0.25 μm, 0.25 mm × 30 m). The column temperature program was as follows: (1) 50 °C for 3 min; (2) temperature was gradually elevated to 220 °C in 17 min; and (3) temperature was gradually elevated to 250 °C in 2 min and maintained for 10 min. Helium was used as the carrier gas, and the flow rate was set to 1.0 mL/min. Electron impact ion source (EI) was applied for Mass spectrometry assay. The temperature was set to 280 °C for injection port, 230 °C for ion source, and 250 °C for the Inlet line. QC samples (pooled sample from an equal aliquot of each sample in the experiment) were injected with SIM mode at the beginning of the MS study and after every five injections throughout the experiment, which was used to monitor the MS performance. Finally, the mass data were analyzed by MSD ChemStation to determine the concentration of each compound.

### Statistics

No statistical methods were used to predetermine the sample sizes. No data exclusions were made in experimental sections and the exclusion criteria in bioinformatics analysis were shown in the corresponding sections. No data show significant deviation from normal distribution and data from different treatment groups show good homogeneity of variances. The exact sample size for each experimental group has been shown in every figure as the number of dots. Lines in each figure represent the mean value of each group. Two-tailed unpaired Student’s *t* test was used to compare the means between two different treatment groups and one-way ANOVA test with post-hoc tests (Tamhane’s T2 multiple comparison test) were used to compare the means among the three or more different treatment groups. All statistical analyses were accomplished with SPSS Statistics (v24.0.0, IBM, USA) and GraphPad Prism (v8.0.1, GraphPad Software, Inc., CA).

## Results

### DOX causes significant death and cardiac dysfunction in mice

**Fig. 1:**
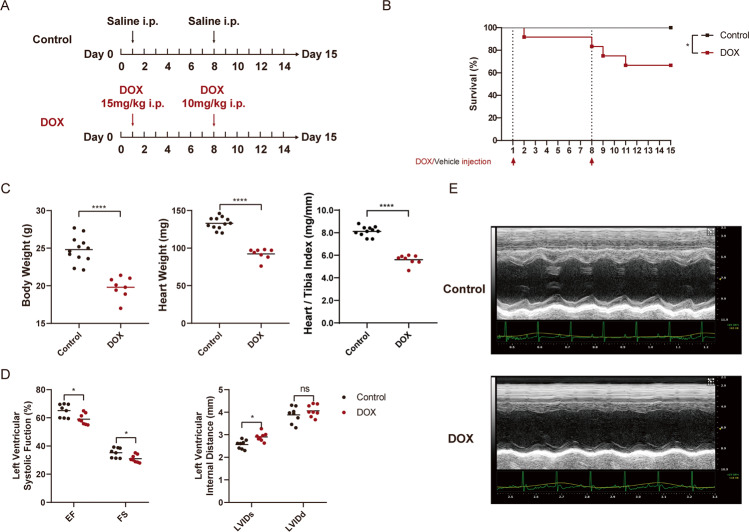
DOX causes significant cardiac dysfunction and death in mice. **a** The protocol of DOX-induced sub-chronic cardiotoxicity model establishment. Mice were treated with saline (vehicle control, *N* = 12) or DOX (*N* = 12) on Day 1 (15 mg/kg DOX, i.p) and Day 8 (10 mg/kg, i.p). **b** Kaplan–Meier survival curves of control and DOX-treated mice during treatment. **c** The heart/body weight ratio was measured in control mice and DOX-treated mice. **d** Echocardiographic analyses of cardiac function in control mice and DOX-treated mice. EF ejection fraction, FS fractional shortening, LVIDs systolic left ventricular internal diameter, LVIDd diastolic left ventricular internal diameter. **e** Representative echocardiograms from control and DOX-treated mice on Day 15. Significance in **b** was calculated using the log-rank (Mantel-Cox) test. Significance in **c**, **d** was calculated using the unpaired Student’s *t* test. *P* value < 0.05 was considered to be significant, and labeled as *P < 0.05; ****P < 0.001; ns not significant.

We first established the DOX-induced sub-acute cardiac injury model using C57/BL6 mice with DOX intraperitoneal injection and measured mice survival for 14 days (Fig. 1a, b). The body and heart weight were measured after killing. As assumed, DOX treatment caused more mice death comparing with vehicle control. DOX-treated mice show lighter body and heart weight, as well as lower Heart/Tibia index comparing with control mice (Fig. 1c). Before euthanasia, the cardiac function of mice was measured with Transthoracic Echocardiography. DOX-treated survived mice demonstrated significant reduction of cardiac systolic function, as suggested by significantly reduced Ejection Fraction (EF), Fractional Shortening (FS), and increased systolic Left Ventricular Internal Diameter (LVIDs) in comparison with control (Fig. 1d, e). These data reveal that DOX causes cardiac injury in mice, mainly affects the systolic function of left ventricular and increases mortality.

### RNA-seq analysis reveals the downregulation of Acot1 dominated biosynthesis of unsaturated fatty acid in DOX-treated murine heart

**Fig. 2:**
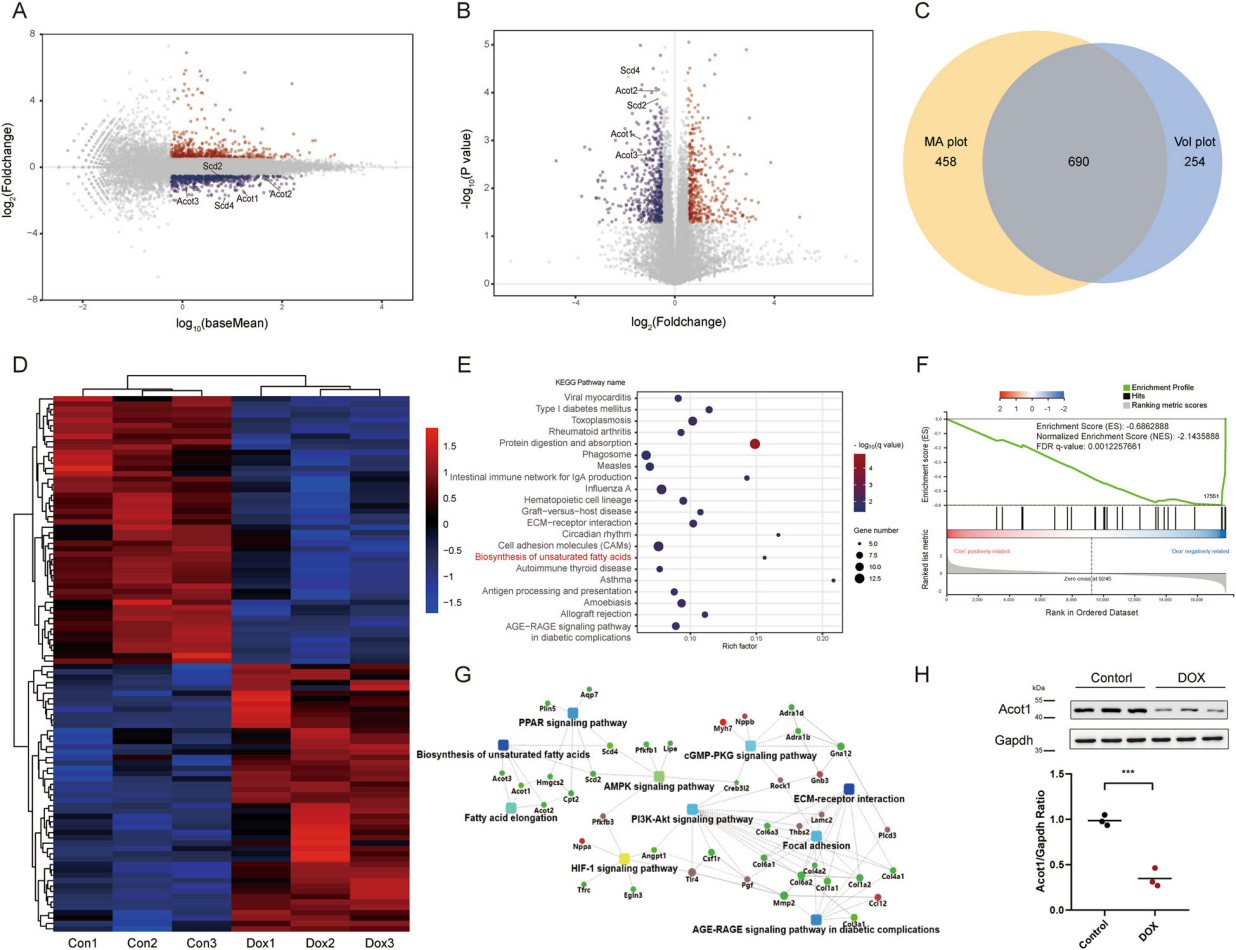
RNA-seq analysis reveals the altered genes in DOX-treated murine heart. **a** MA plot representing regulated genes with higher base expression. **b** Volcano plot representing regulated genes with fold change. Red and blue plot dots mean the increased or decreased expression in the DOX-treated murine heart comparing with the control murine heart in **a, b**. Labeled dots representing significant regulated genes involved in the selected pathway. **c** Venn diagram representing the screened genes in the intersection part with **a, b**. **d** Heat map representing the most significantly regulated genes detected in RNA-seq analysis with control and DOX-treated murine heart tissue. Gene expressions were normalized with row *Z*-score. **e** KEGG pathway enrichment analysis in DOX-treated murine heart comparing with control murine heart. **f** Gene set enrichment analysis (GSEA) of regulated genes in DOX-treated murine heart comparing with control murine heart. **g** Protein–Protein interaction network analysis with the significantly regulated genes. **h** Western blot confirming the change of Acot1 in the DOX-treated murine heart. Significance in **h** was calculated using the unpaired Student’s *t* test. *P* value < 0.05 was considered to be significant, and labeled as ***P < 0.005.

RNA-sequencing (RNA-seq) analysis was performed to determine the differentially expressed genes using DOX-treated and control murine hearts, to further explore the potential molecular mechanisms. MA-plot and Volcano-plot were performed to identify the significantly regulated genes with a middle-high expression basement (Fig. 2a, b). The genes in the intersection were then used for enrichment analysis. (Fig. 2c). The most regulated genes, with 50 up-regulated and 50 down-regulated, show good consistency in each sample (Fig. 2d). Expanded lists of differentially expressed genes were provided (Supplementary Tables 1 and 2). In KEGG-pathway enrichment analysis, the biosynthesis of unsaturated fatty acids was one of the most significantly down-regulated metabolic pathways, which containing Acot1, Acot2, Acot3, Scd2, and Scd4 (Fig. 2e). GSEA analysis also confirmed the same pathway negatively related enriched after DOX treatment and Acot1 as one of the leading-edge core genes in the gene set (Fig. 2f). Protein–Protein Interaction (PPI) network analysis was performed with the significantly changed genes (Fig. 2g). Real-time PCR showed that DOX treatment led to a significant decrease in mRNA levels of listed genes (Supplementary Fig. 1). Moreover, the protein level of Acot1 was then confirmed by western blot (Fig. 2h).

Dysregulation of iron metabolism has long been considered to be the primary mechanism that causes cardiomyocytes death in DOX-induced cardiac injury^28^. Taken iron metabolism and biosynthesis of polyunsaturated fatty acid together, a novel pattern of cell death, ferroptosis, come into our mind. Though identified as a novel form of regulated cell death that results from iron-dependent lipid peroxidation, the molecular mechanism of ferroptosis has not yet been fully elucidated, and few studies have been done in cardiovascular diseases^29^. Our subsequent studies then confirmed the existence of ferroptosis in this pathological process. Surprisingly, the RNA-seq analysis did not show significant enrichment in ferroptosis or iron metabolism-related bio-pathways that we currently have known (Supplementary Table 3).

### Ferroptosis may participate in the cardiotoxicity induced by DOX

**Fig. 3:**
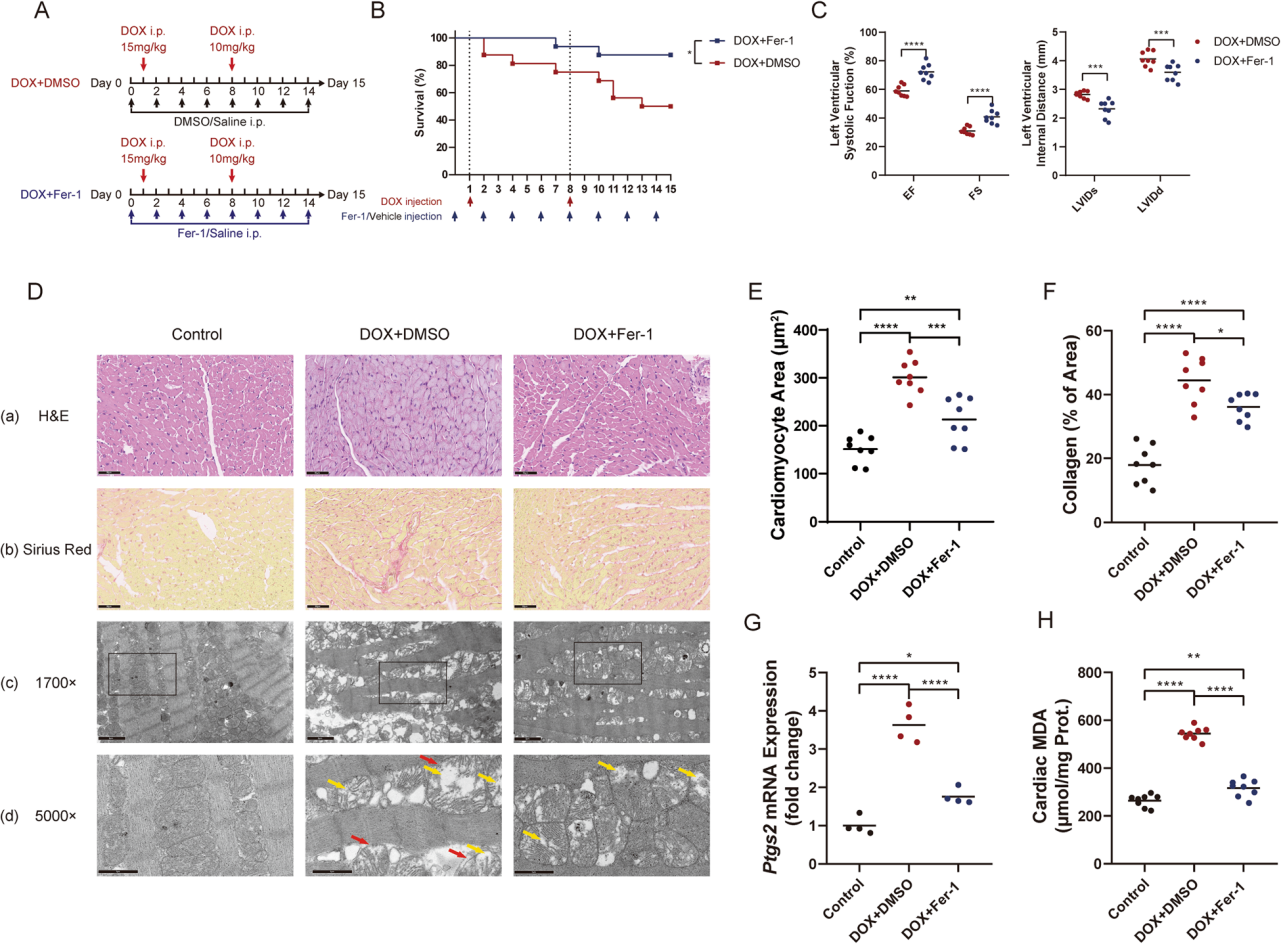
Ferroptosis may be related to the cardiotoxicity induced by DOX. **a** The protocol of testing the protective effect of Fer-1 in the DOX-induced sub-chronic cardiotoxicity model. Mice were all treated with DOX on Day 1 (15 mg/kg DOX, i.p) and Day 8 (10 mg/kg, i.p). Fer-1 (1 mg/kg, *N* = 16) or DMSO (vehicle control, *N* = 16) dissolve in saline were administrated every other day from Day 0. **b** Kaplan–Meier survival curves of Fer-1 treated and control mice during DOX treatment. **c** Echocardiographic analyses of cardiac function in control mice and Fer-1-treated mice. EF ejection fraction, FS fractional shortening, LVIDs systolic left ventricular internal diameter, LVIDd diastolic left ventricular internal diameter. **d** Representative H&E staining (**a**) and Sirius red staining (**b**) images in the heart tissue sections of control mice and DOX-treated mice with Fer-1 or DMSO (vehicle control). Representative transmission electron microscopy images in ×1700 (**c**) and ×5000 (**d**). Yellow arrow showing the vacuolization of mitochondrial; red arrow showing the rupture of the outer mitochondrial membrane. **e** Quantitive analysis of cardiomyocyte area in sections of control mice and DOX-treated mice with Fer-1 or DMSO. **f** Quantitative analysis of collagen area percentage in sections of control mice and DOX-treated mice with Fer-1 or DMSO. **g** Ptgs2 mRNA level in control mice and DOX-treated mice with Fer-1 or DMSO. **h** Cardiac MDA level (relative to protein concentration) in control mice and DOX-treated mice with Fer-1 or DMSO. Significance in **b** was calculated using the log-rank (Mantel–Cox) test. Significance in **c** was calculated using the unpaired Student’s *t* test. Significance in **e**–**h** was calculated using the one-way ANOVA test with multiple comparisons between the two groups. *P* value < 0.05 was considered to be significant, and labeled as *P < 0.05; **P < 0.01; ***P < 0.005; ****P < 0.001.

To further explore the influence of ferroptosis in DOX-induced cardiotoxicity, Fer-1, a selective small molecular inhibitor of ferroptosis, was used to test the cardiac protective function of ferroptosis inhibition in mice treated with DOX. DOX-induced sub-acute cardiac injury model was established with or without Fer-1 intraperitoneal injection every other day (Fig. 3a). Fer-1 treated mice demonstrated a lower death rate and preserved cardiac function (Fig. 3b, c). We then tested the histological changes in DOX-treated murine heart tissue. Comparing with control mice and Fer-1 treated mice, DOX-treated myocardium tissue indicated obvious lipofuscin deposition after 14 days, accompanied by an enlarged cross-sectional area due to cardiomyocyte edema (Fig. 3d(a), e and Supplementary Fig. 2a). Also, extensive myocardial fibrosis was observed in the DOX-treated murine heart (Fig. 3d(b), f). To further validate the existence of ferroptosis, electron transmission microscopy was used to detect the ultrastructure changes of cardiomyocytes. DOX-induced significant myolysis, myofilament loss, and disarrangement in cardiomyocytes, while Fer-1 treatment alleviated these pathological changes (Fig. 3d(c), (d)). Besides, serum cardiac troponin I (cTnI) was used to evaluate the cardiac injury level (Supplementary Fig. 2B).

Meanwhile, Ptgs2 mRNA expression and cardiac MDA level, both seen as potential ferroptosis signature markers, were up-regulated by DOX administration (Fig. 3g, h). We then tested the changes of some ferroptosis-related molecules, including Gpx4, Acsl4, and Fsp1 (Supplementary Fig. 2C). No significant differences were seen among groups.

While apoptosis induced by doxorubicin administration has long been considered as the primary pattern of regulated cell death, which lead to the loss of the cardiomyocytes, we tested the expression level of some apoptosis-related genes. We also explored the changes after the inhibition of ferroptosis. Decreased P53/Gapdh ratio and increased Bax/Bcl2 ratio, Cleaved Caspase-3/Gapdh ratio, and Cleaved Parp1/Gapdh ratio was seen in DOX-treated mice. Fer-1 co-treatment reversed these changes partially. The Caspase-8/Gapdh ratio does not show a significant difference among groups (Supplementary Fig. 3).

These results show that the inhibition of ferroptosis during DOX treatment preserves cardiac function, providing additional evidence that the ferroptosis is involved in this pathological process. Interestingly, inhibition of ferroptosis may also protect cardiomyocytes from apoptosis.

### DOX causes ferroptotic-like cell death in HL-1 cardiomyocytes and a reduction of Acot1 expression

**Fig. 4:**
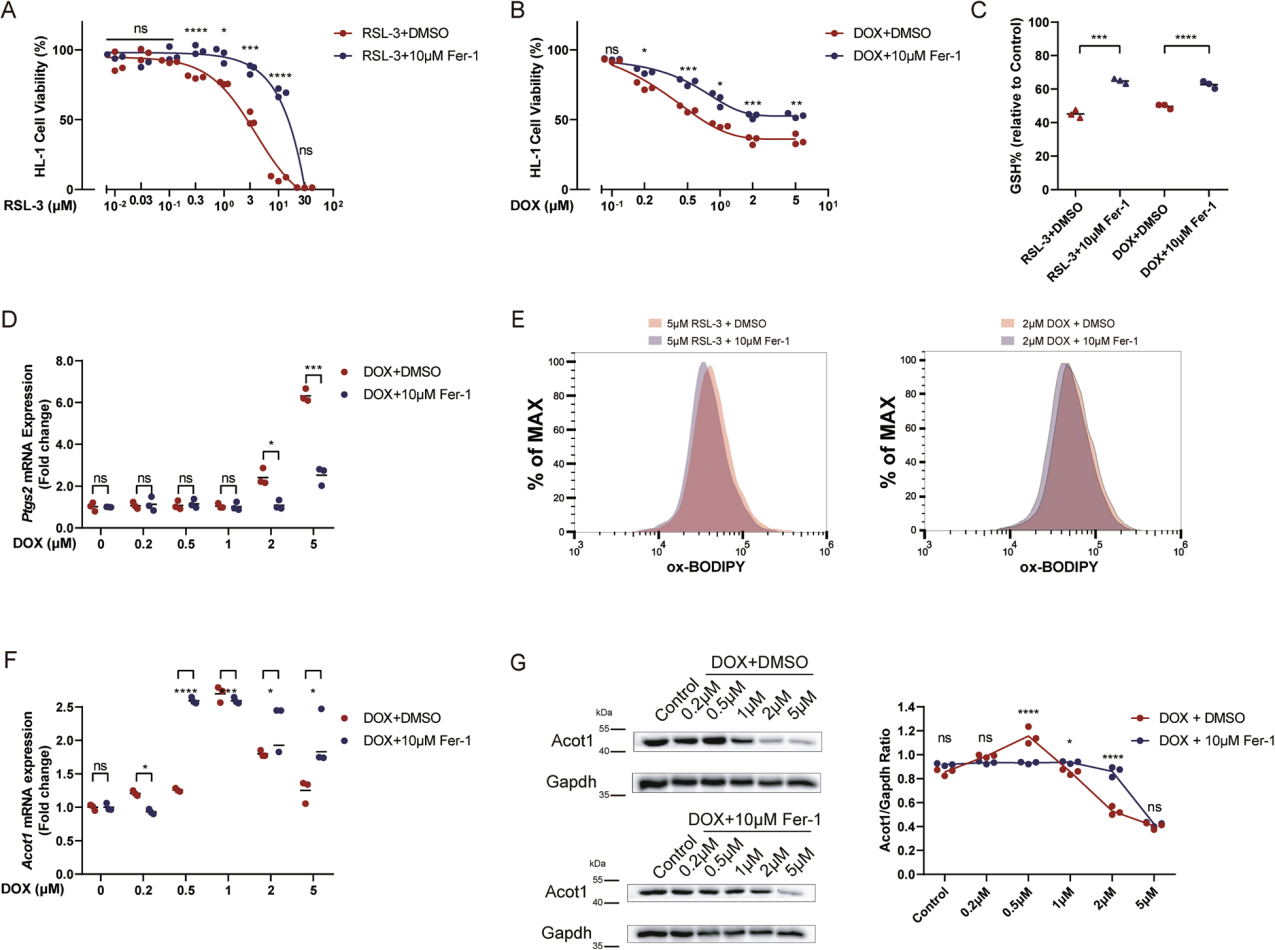
DOX induces ferroptotic-like cell death in HL-1 cardiomyocytes and decreases Acot1 expression. **a** HL-1 Cell viability analysis of the protective effect of Fer-1 (10 μM) in different concentrations of RSL-3-induced cell death for 24 h. **b** HL-1 Cell viability analysis of the protective effect of Fer-1 (10 μM) in different concentrations of DOX-induced cell death for 24 h. **c** Relative intracellular GSH/GSSG levels in RSL-3 (5 μM) or DOX (2 μM) treated HL-1 cells with or without Fer-1 (10 μM) for 6 h. **d** Ptgs2 mRNA level in different concentrations of DOX-treated HL-1 cell with or without Fer-1 (10 μM) for 24 h. **e** Flow cytometer analysis of C11-BODIPY 581/591 staining showing lipid peroxidation level in RSL-3 (5 μM) or DOX (2 μM) treated HL-1 cells with or without Fer-1 (10 μM) for 6 h. **f** Acot1 mRNA levels in different concentrations of DOX-treated HL-1 cell with or without Fer-1 (10 μM) for 24 h. **g** Acot1 protein levels in different concentrations of DOX-treated HL-1 cell with or without Fer-1 (10 μM) for 24 h. Significance in **a-d**, **f**, **g** was calculated using the unpaired Student’s *t* test. *P* value < 0.05 was considered to be significant, and labeled as *P < 0.05; **P < 0.01; ***P < 0.005; ****P < 0.001; ns not significant.

After validating the induction of ferroptosis in DOX-treated mice, we next investigated how Acot1 influenced the process in cardiomyocytes. RSL-3, a class II small molecular inducer of ferroptosis, was used to establish the ferroptosis model in HL-1 cell lines^30,31^. We evaluated the cytotoxic ability of RSL-3 in a concentration gradient with or without Fer-1. Also, the DOX-induced cardiomyocyte death model was established using a similar method. A significant increase of cell death by DOX and RSL-3 administration was detected in a dose-dependent way, while Fer-1 co-treatment inhibited the tendency (Fig. 4a, b). We then measured glutathione levels (GSH and GSSG) in HL-1 cells treated with RSL-3 and DOX. The ratio of GSH/total glutathione showed that both RSL-3 and DOX treatment decreased the antioxidant ability of cardiomyocytes (Fig. 4c). DOX treatment could induce ferroptosis as verified by the elevation of *Ptgs2* mRNA level in 2 μM, and 5 μM DOX-treated HL-1 cells, while Fer-1 co-treatment inhibited the elevation of *Ptgs2* (Fig. 4d). Lipid peroxidation measured with C11-BODIPY 581/591 probe was recognized as the gold standard for the presence of ferroptosis. As demonstrated, both RSL-3 and DOX treatment caused the significant generation of lipid ROS, which could be inhibited by Fer-1 co-treatment (Fig. 4e). Therefore, Fer-1 treatment may inhibit ferroptosis via anti-lipid peroxidation ability and subsequently decrease DOX caused cell death. Moreover, DOX induces cytotoxicity in HL-1 cells in a pattern similar to RSL-3 induced ferroptosis cell death. We finally tested the influence of DOX treatment on the Acot1 expression. As shown, Acot1 expression was decreased at a higher concentration of DOX, while Fer-1 co-treatment partially suppressed this trend (Fig. 4f, g). Thus, we propose that Acot1 may be a potential protective factor of ferroptosis.

### Acot1 knock-down sensitizes cardiomyocytes to DOX toxicity caused by ferroptosis

**Fig. 5:**
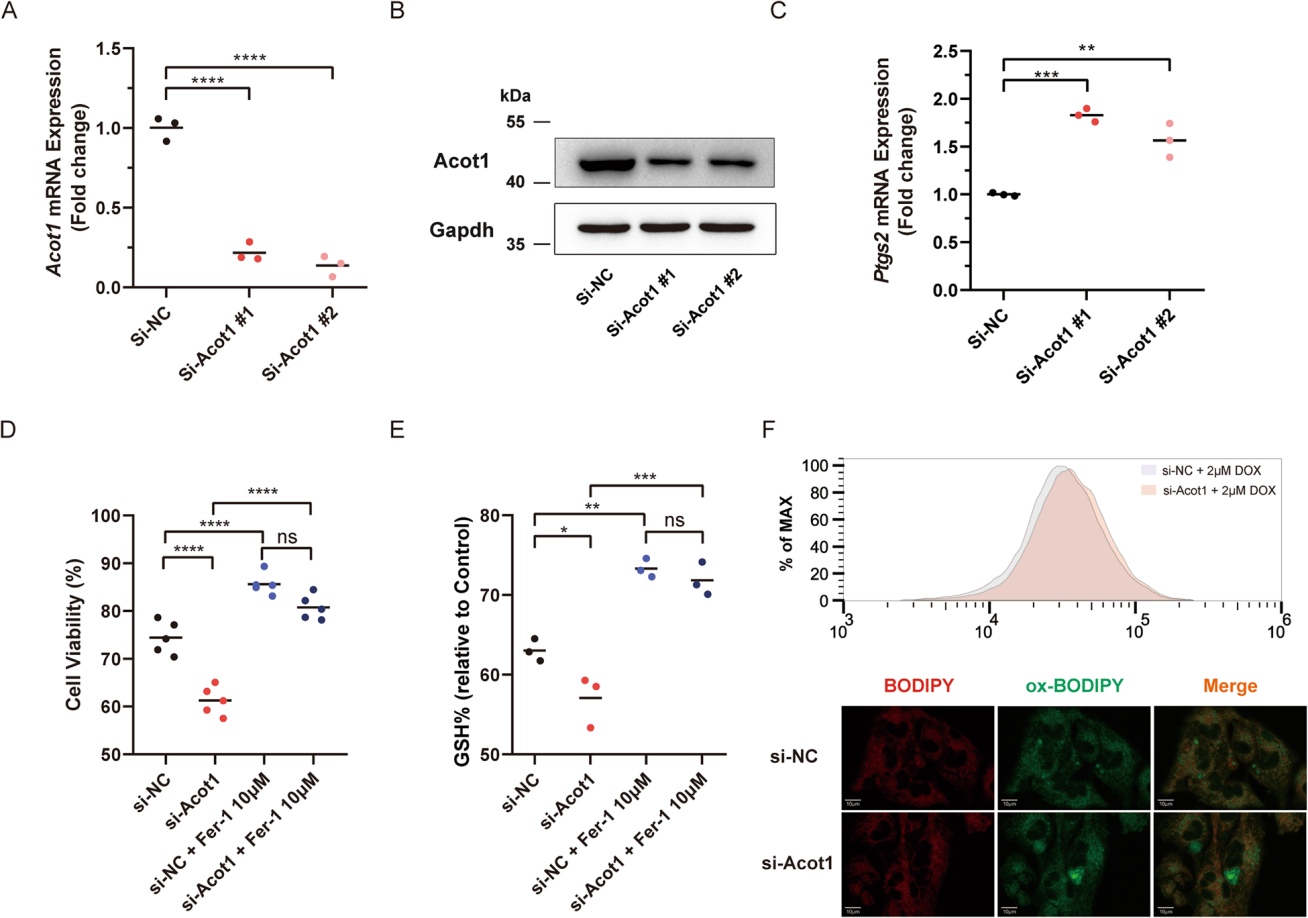
Acot1 knock-down sensitized cardiomyocytes to DOX toxicity caused by ferroptosis. **a** Realtime-qPCR confirming the efficiency of si-Acot1 #1, #2 comparing with scrambling si-NC. **b** Western blot confirming the efficiency of si-Acot1 #1, #2 comparing with scrambling si-NC. **c** Ptgs2 mRNA level in DOX (2 μM, 24 h) treated HL-1 cells transfected with si-Acot1 #1, #2, and si-NC. **d** Cell viability analysis showing the effect of Acot1 knock-down in DOX (2 μM, 24 h) induced cell death and the protective effect of Fer-1 (10 μM) co-treatment. **e** The intracellular GSH/GSSG levels showing the effect of DOX (2 μM, 6 h) treated HL-1 cells with or without Acot1 knock-down and the protective effect of Fer-1 (10 μM) co-treatment. **f** Flow cytometer analysis of C11-BODIPY 581/591 staining showing lipid peroxidation level in DOX (2 μM, 6 h) treated HL-1 cells with or without Acot1 knock-down and the representative fluorescent images showing lipid peroxidation level. Significance in **a**, **c** was calculated using the unpaired Student’s *t* test. Significance in **d, e** was calculated using the one-way ANOVA test with multiple comparisons between two groups. *P* value < 0.05 was considered to be significant, and labeled as *P < 0.05; **P < 0.01; ***P < 0.005; ****P < 0.001; ns not significant.

As reported before, Acot1 serves as a primary player in the catalytic hydrolysis of acyl-CoA to free fatty acids and CoA-SH^11^. Acsl4, a functionally opposite enzyme of Acot1 in lipid metabolism that catalyzes the synthesis of arachidonyl-CoA, was reported to play a role in the induction of ferroptosis^32^. Considering the reduced expression of Acot1 by DOX shown in our study, we think that Acot1 may have the potential ability to inhibit cell ferroptotic death. To verify it, we then examined whether knock-down Acot1 influenced the sensitivity of HL-1 cells to DOX toxicity. The interference efficiency of each siRNA was determined by real-time PCR and western blot independently, and Si-Acot1 #1 was selected for the further experiment (Fig. 5a, b). Real-time PCR show an elevated *Ptgs2* mRNA level in Acot1 knock-down cardiomyocytes treated with DOX (Fig. 5c). Cell viability assay showed that reduced expression of Acot1 increased cell death induced by DOX treatment, which still could be rescued by Fer-1 co-treatment (Fig. 5d). Decreased GSH/total glutathione ratio was also observed in Acot1 knock-down cells comparing with scramble control (Fig. 5e). Acot1 knock-down caused more lipid peroxidation as tested by C11-BODIPY staining after DOX treatment (Fig. 5f). These data demonstrated enhanced sensitivity to DOX toxicity after Acot1 knock-down in cardiomyocytes.

### Acot1 overexpression inhibits lipid peroxidation and DOX cytotoxicity

**Fig. 6:**
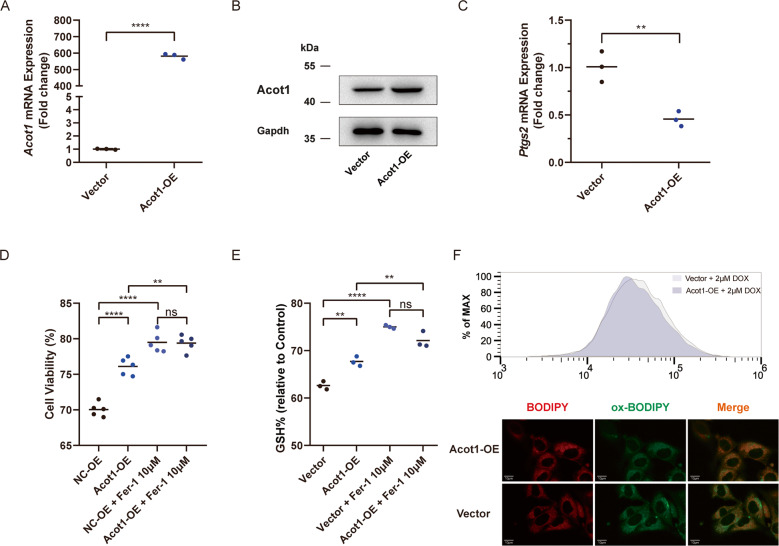
Acot1 overexpression inhibits lipid peroxidation and DOX cytotoxicity. **a** Realtime-qPCR confirming the efficiency of Acot1-OE plasmid comparing with vector control. **b** Western blot confirming the efficiency of Acot1-OE plasmid comparing with vector control. **c** Ptgs2 mRNA level in DOX (2 μM, 24 h) treated HL-1 cells transfected with Acot1-OE and vector. **d** Cell viability analysis showing the effect of Acot1 overexpression in DOX (2 μM, 24 h) induced cell death and the protective effect of Fer-1 (10 μM) co-treatment. **e** The intracellular GSH/GSSG levels showing the effect of DOX (2 μM, 6 h) treated HL-1 cells with or without Acot1 overexpression and the protective effect of Fer-1 (10 μM) co-treatment. **f** Flow cytometer analysis of C11-BODIPY 581/591 staining showing lipid peroxidation level in DOX (2 μM, 6 h) treated HL-1 cells with or without Acot1 overexpression and the representative fluorescent images showing lipid peroxidation level. Significance in **a**, **c** was calculated using the unpaired Student’s *t* test. Significance in **d, e** was calculated using the one-way ANOVA test with multiple comparisons between the two groups. *P* value < 0.05 was considered to be significant, and labeled as **P < 0.01; ****P < 0.001; ns not significant.

Next, we wonder whether the augmentation of Acot1 expression may reduce the cytotoxicity by DOX. Acot1 overexpression (Acot1-OE) plasmid and vehicle vector were individually transfected into cardiomyocytes. The elevated expression of Acot1 mRNA and protein were verified by comparing Acot1-OE plasmid transfected cell with vector control (Fig. 6a, b). Declined Ptgs2 mRNA level was observed in Acot1 overexpression cardiomyocytes treated with DOX as compared with control (Fig. 6c). Cell death induced by DOX was inhibited by Acot1 overexpression, and Fer-1 treatment still could rescue the cell death induced by DOX (Fig. 6d). Comparing with vector control, Acot1 overexpression elevated GSH/total glutathione ratio, indicating decreased oxidative stress and reserved antioxidant capacity (Fig. 6e). Consistent with the rescue of cell death, Acot1 overexpression inhibited lipid peroxidation in cardiomyocytes, as verified by C11-BODIPY 581/591 staining (Fig. 6f). Altogether, these data show that Acot1 plays an important protective role in the process of DOX caused cell death.

### αMHC-Acot1 transgenic mice show altered free fatty acid composition

**Fig. 7:**
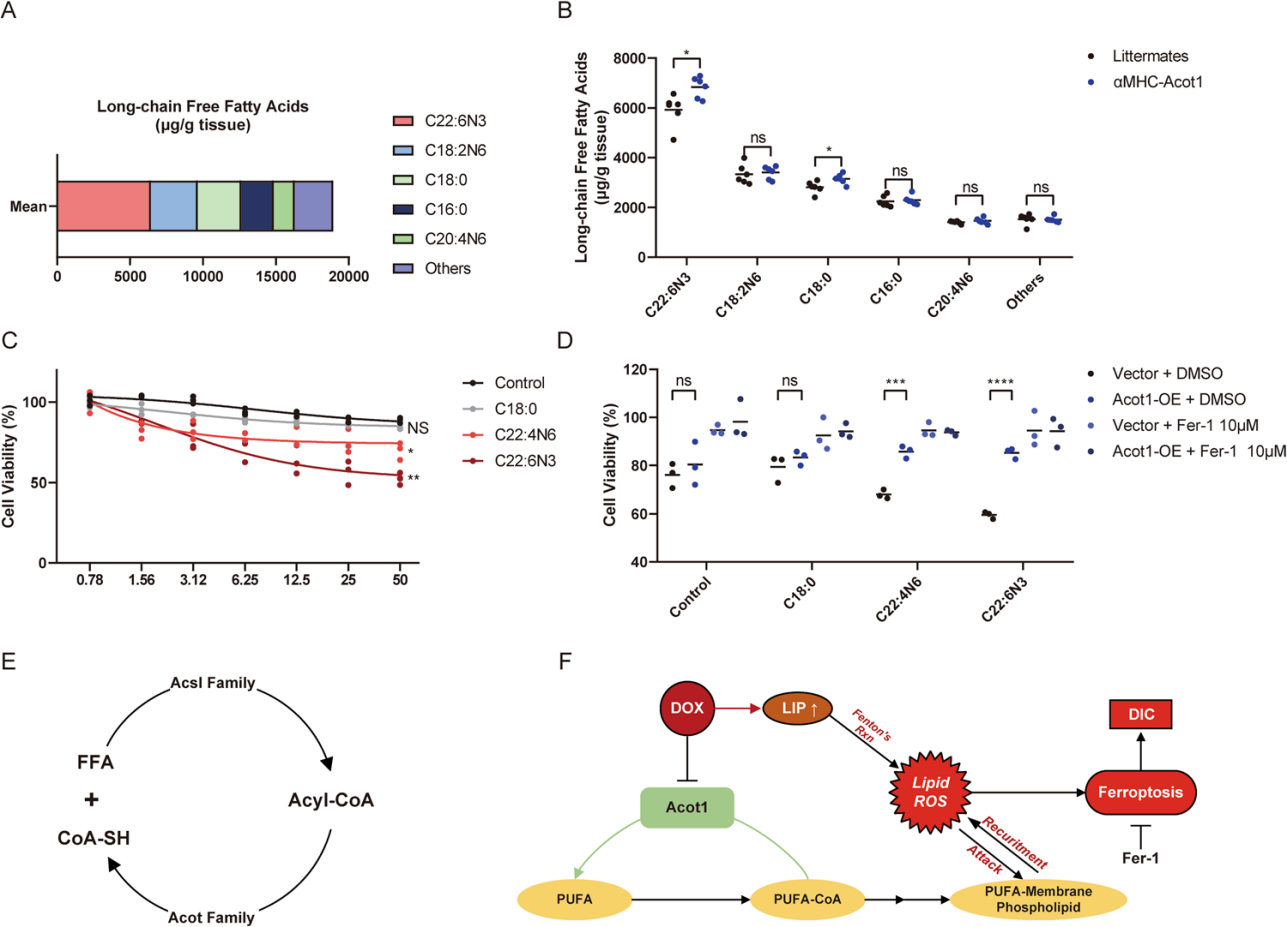
αMHC-Acot1 transgenic mice show reshaped free fatty acid composition. **a** The mean concentrations of free fatty acids detected by GC-MS in αMHC-Acot1 transgenic mice and their littermates. **b** Top 5 FFAs concentrations in murine heart tissue. **c** Cell viability analysis showing different concentrations of FFAs enhanced DIC in HL-1 cells. **d** Cell viability analysis showing the protective effect of Acot1 overexpression and Fer-1 co-treatment to FFAs enhanced DIC. **e** Diagrammatic method of showing the opposite enzymatic function of Acsl family and Acot family in the cell. **f** The potential mechanism of Acot1 inhibits the induction of ferroptosis in DOX-induced cardiotoxicity. LIP liable iron pool, DIC doxorubicin-induced cardiotoxicity, Fer-1 Ferrostatin-1. Significance in **b** was calculated using the unpaired Student’s *t* test. Significance in **c**, **d** was calculated using the one-way ANOVA test with multiple comparisons between the two groups. *P* value < 0.05 was considered to be significant, and labeled as *P < 0.05; **P < 0.01; ***P < 0.005; ****P < 0.001; ns not significant.

Considering the biochemical function of Acot1 and Acsl4, we proposed that the protective effect of Acot1 was due to its ability to shape lipid composition in cardiomyocytes. As reported before, certain kinds of unsaturated fatty acid sensitized cells to ferroptosis^33^. Therefore, Gas chromatography-mass spectrometry (GC-MS) was used to detect the free fatty acid composition in αMHC-Acot1 transgenic mice and their littermates. Generally, murine heart tissue contains abundant free fatty acids, due to its energy metabolic preference (Fig. 7a). Acot1 transgenic murine heart tissues show significantly elevated concentrations of C22:6N3 (Docosahexaenoic Acid, DHA) and C18:0 (Stearic acid), but none significance in C18:2N6 (Linoleic acid), C16:0 (palmitic acid) and C20:4N6 (arachidonic acid, AA) concentrations (Fig. 7b). We then tested the free fatty acids induced sensitization of DIC in HL-1. Data showed a decreased cell viability with a gradient of FFA concentration, and DHA treatment significantly sensitized HL-1 cells to DIC (Fig. 7c). While Fer-1 co-treatment showed a protective effect, Acot1 overexpression also desensitized cardiomyocytes to DHA and AA enhanced DIC (Fig. 7d). Thus, the protective role of Acot1 in DIC was possibly due to the enzymatic function that regulates the free fatty acid composition and distribution in cardiomyocytes, particularly DHA, and then induces desensitization of cardiomyocytes to ferroptosis.

## Discussion

Regulated cell death in terminally differentiated cardiomyocytes plays an important role and has been one of the major interests of investigation in many cardiac pathologies, including DIC^32,34^. Results that inhibition of any one of previously described mechanisms failed to inhibit DIC completely indicate the presence of other unrevealed mechanisms in DIC, which may be related to the dysregulation of iron homeostasis and oxidative stress^3,35,36^. Recently, a research group creatively demonstrated the presence of ferroptosis in the DIC process^10^.

Ferroptosis, as a new form of cell death discovered in 2012, results from iron-dependent lipid peroxide accumulation^7^. While still in its early era, its complete molecular mechanism and its role in disease have remained to be elucidated. Till now, the best-known mechanisms that have been related to ferroptosis were: (1) Excess lipid peroxidation induced by Fenton’s reaction (iron-catalyzed oxidative reaction) or lipoxygenase family^33,37^; (2) Lipid hydroperoxides accumulation caused by dysfunction of System x_c_^−^/Gpx4 system^30,38^.

Researches in ferroptosis mainly focused on the pathological process of the tumor, yet few of them promoting the important role of ferroptosis in the cardiac disease. In a study of myocardial infarction, the transcription factor BTB domain and CNC homolog 1 (BACH1) regulate and further influence a set of genes that attenuate ferroptosis, including glutamate-cysteine ligase modifier subunit (Gclm), ferritin heavy chain 1 (Fth1), ferritin light chain 1 (Ftl1), solute carrier family 7 member 11 (Slc7a11), and solute carrier family 40 member 1 (Slc40a1)^39^. In another study of DIC, Nrf2/Hmox1 axis has been considered as a pro-ferroptosis factor, due to their ability to rapid conversion heme to free iron and thereby induce iron accumulation in cardiac tissue^10^. These findings encourage us to further explore the importance and relevant mechanisms of ferroptosis in cardiac diseases, such as DIC.

We firstly tested and proved the presence and value of ferroptosis in DIC. As shown in the study, ferroptotic-like morphological changes, characterized by the reduction or vanishing of mitochondria crista and outer mitochondrial membrane rupture^40^, were observed in the DOX-treated murine heart with transmission electron microscopy. Biochemical features of ferroptosis including increased lipid peroxidation was also confirmed by increased MDA concentration and by elevated *Ptgs2* mRNA level^41^. Moreover, DOX-treated mice presented reduced cardiac systolic function and increased death rate, while Fer-1 co-treatment reduced the cardiotoxicity and mortality by DOX. Fer-1 is a small molecular inhibitor of ferroptosis, and its anti-ferroptotic activity is related to the scavenging of ferrous iron-initiated lipid hydroperoxides^42^. The protective effect of Fer-1 to DIC also suggests that ferroptosis may be one of the fundamental mechanisms in the process.

We then wondered what would be the core player of ferroptosis. Some well-known modulators of ferroptosis were tested. Gpx4 and FSP1 (previously known as apoptosis-inducing factor mitochondrial 2 (Aifm2)) were both identified as ferroptosis suppression factors, while Acsl4 was found to induce the sensitivity to ferroptosis^30,43–45^. However, no significant differences in these genes were seen among groups. A previous study has pointed out that acyl-CoA synthetase long-chain family member-4 (Acsl4) was required for ferroptosis activation. At the same time, Acsl4 deficiency shows great resistance to ferroptosis and significantly reduces the formation of lipid-ROS^46^. The role of ACSL4 in the ferroptotic cell death process rests upon its ability to ligate coenzyme A to long-chain PUFAs, especially with a preference of arachidonic acid (AA) and adrenic acid (AdA), dictate ferroptosis sensitivity by shaping cellular membrane lipid composition^33^. Thus, we speculated that genes modifying the composition of PUFA in the membrane lipids might manipulate the sensitivity to the cell damage caused by ferroptosis inducers as doxorubicin.

The transcriptome sequencing and bioinformatics analysis in the study proved our speculation. With the pathway enrichment analysis and GSEA after RNA-sequencing in the current study, significant downregulation was observed in the pathway of biosynthesis of unsaturated fatty acid in the DOX-treated murine heart. In the sub-acute phase after DOX administration, the protective mechanisms in cardiomyocytes were thought to be inhibited. The PPI-analysis including Gene Ontology (GO) annotation did not screen out a significant hub-gene but provided clues for the possible bio-pathways involved in the pathological process with the most regulated genes. As mentioned in other studies of DIC, the AMPK signaling pathway was also down-regulated in the DOX-treated murine heart and therefore is a potential protective target^47^. Comparing with Scd4, Acot1 has a higher base expression level and mainly localized in the cytosol of cells. Considering the likely mechanism in ferroptosis and an essential role of Acsl4 in it, we then chose Acot1, which catalyze the opposite molecular process of Acsl4, as our major target (Fig. 7e). Moreover, our previous studies have figured out that Acot1 may cast a cardioprotective effect in the process of LPS-induced cardiomyopathy and diabetic cardiomyopathy by decreasing the oxidative stress in cardiomyocytes.

Results from the study by overexpression of Acot1 or knock-down Acot1 verified the beneficial role of Acot1 in DIC. Our previous studies have reported that Acot1 overexpression prevents cardiac dysfunction in both LPS-induced cardiomyopathy and diabetic cardiomyopathy by reducing oxidative stress^14,15^. Different from them, our current data demonstrated that Acot1 might cast a protective effect in DIC via inhibition of ferroptosis.

Unlike the preferences of Acsl4 in lipid metabolism, an elevated concentration of DHA in the heart tissues of αMHC-Acot1 transgenic mice was observed in the study, yet still as potential substrates for causing ferroptosis. We then confirmed that DHA treatment could sensitize cells to DIC. Surprisingly, we also found that Acot1 overexpression in vitro reduced cell death by DHA. The conflict of exogenous free DHA sensitizes cardiomyocytes to ferroptosis, while Acot1 increases DHA level but prevents DIC may be explained as the augmented PUFA ratio in the bio-membrane structure. The whole process may be regulated by a series of lipid metabolism enzymes, including the synthesis of DHA-CoA and the insertion of DHA-CoA into the bio-membrane^9,46^. In line with the findings in Acot1 transgenic murine heart tissue, Acot1 overexpression in cardiomyocytes may interrupt the regulated process and induce the alteration of lipid composition in cardiomyocytes. Thus, the protective effect of Acot1 on ferroptosis may derive from the altered bio-membrane fatty acids composition of cardiomyocytes in DIC.

Additionally, other lipid metabolism-related genes screened in the current study may also play an important role in the DIC process. Stearoyl-CoA desaturase (Scd) family catalyze the desaturation of saturated acyl-CoA, and Scd1 was nearly reported to be a protective factor in ovarian cancer cells from ferroptotic cell death induced by RSL-3^48,49^. The role of Scd family, specifically Scd4 and Scd2, in the induction of DIC needs further investigation.

What is noteworthy is that ferroptosis does perform an important, but not the only player in the pathology process of DIC. Other than the ROS-based mechanism, Top2β in cardiomyocytes may also be a susceptible candidate to DIC^5^. Ferroptosis inhibition can but only partially rescue the cardiotoxicity of DOX in both in vitro and in vivo experiments. Our data show Fer-1 treatment not only rescue cardiomyocytes from ferroptotic cell death but also reduced the induction of cardiomyocytes apoptosis. To our best knowledge, the process of DIC contains various cell death mechanisms, including but not limited to autophagy, senescence, and pyroptosis. The current work confirmed the presence of ferroptosis in the net-linked mechanisms of DIC, while the cross-talk between these mechanisms still need a series of further investigations.

In conclusion, our study provides evidence suggesting the protective effect of Acot1 in the process of DIC. The underlying mechanism may be related to the reshaping of free fatty acids composition by Acot1 and subsequently desensitization of cardiomyocytes to ferroptosis (Fig. 7f). Further studies will be needed to verify whether Acot1 is a suitable protective target in DIC.

## Supplementary information

Supplementary Figure legends

Supplementary Figure 1

Supplementary Figure 2

Supplementary Figure 3

Supplementary Table 1

Supplementary Table 2

Supplementary Table 3

Supplementary Table 4
