# Supplementary material for: Acyl-CoA thioesterase 1 prevents cardiomyocytes from Doxorubicin-induced ferroptosis via shaping the lipid composition
Source: Cell Death Dis. 2020 Sep 15;11(9):756. doi: 10.1038/s41419-020-02948-2 (PMC7492260; doi:10.1038/s41419-020-02948-2)
Supplement: Supplementary file 1 — Supplementary Figure legends [file 41419_2020_2948_MOESM1_ESM.docx]

**Supplementary Fig.1 DOX decreases genes related to unsaturated fatty acid biosynthesis.**

(A) - (E) Acot1, Acot2, Acot3, Scd2, and Scd4 mRNA levels in control mice and DOX treated mice.

Significance in (A)–(E) was calculated using the One-Way ANOVA test with multiple comparisons between the two groups. P value<0.05 was considered to be significant, and labeled as ***P < 0.005; ****P < 0.001.

**Supplementary Fig.2 Fer-1 reduces DOX-induced cardiotoxicity.**

(A) Representative WGA staining images in the heart tissue sections of control mice and DOX treated mice with Fer-1 or DMSO (vehicle control). (B) Serum Troponin I level of control mice and DOX treated mice with Fer-1 or DMSO, (C) Ferroptosis related molecules expression level detected by western blot. (D) - (F) Acsl4/Gapdh, Gpx4/Gapdh, Fsp1/Gapdh expression level in control mice and DOX treated mice with Fer-1 or DMSO.

Significance in (B), (D)-(F) was calculated using the One-Way ANOVA test with multiple comparisons between the two groups. P value<0.05 was considered to be significant and labeled as *P < 0.05; **P < 0.01; ns, not significant.

**Supplementary Fig.3 Fer-1 partly inhibits DOX-induced apoptosis.**

(A) Apoptosis related molecules expression level detected by western blot. (B)-(F) Ratio of Bax/bcl2, P53/Gapdh, Cleaved Parp1 / Gapdh, Cleaved caspase-8 / Gapdh, Cleaved caspase-3 / Gapdh expression level in control mice and DOX treated mice with Fer-1 or DMSO.

Significance in (B)-(F) was calculated using the One-Way ANOVA test with multiple comparisons between two groups. P value<0.05 was considered to be significant, and labeled as *P < 0.05; **P < 0.01; ****P < 0.001; ns, not significant.
