# Supplementary figures and images for: Acyl-CoA thioesterase 1 prevents cardiomyocytes from Doxorubicin-induced ferroptosis via shaping the lipid composition
Source: Cell Death Dis. 2020 Sep 15;11(9):756. doi: 10.1038/s41419-020-02948-2 (PMC7492260; doi:10.1038/s41419-020-02948-2)

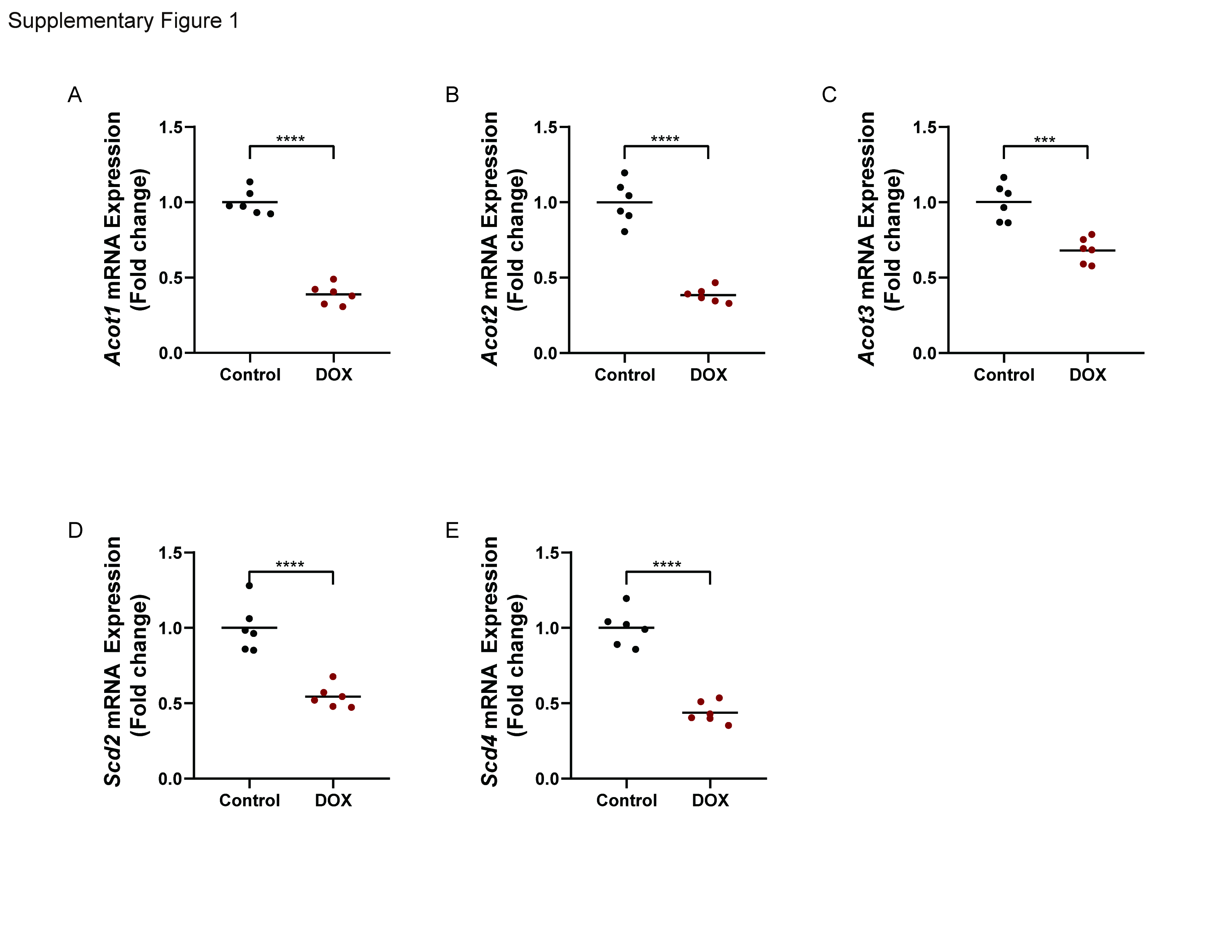

Supplement: Supplementary file 2 — Supplementary Figure 1 [file 41419_2020_2948_MOESM2_ESM.tif]

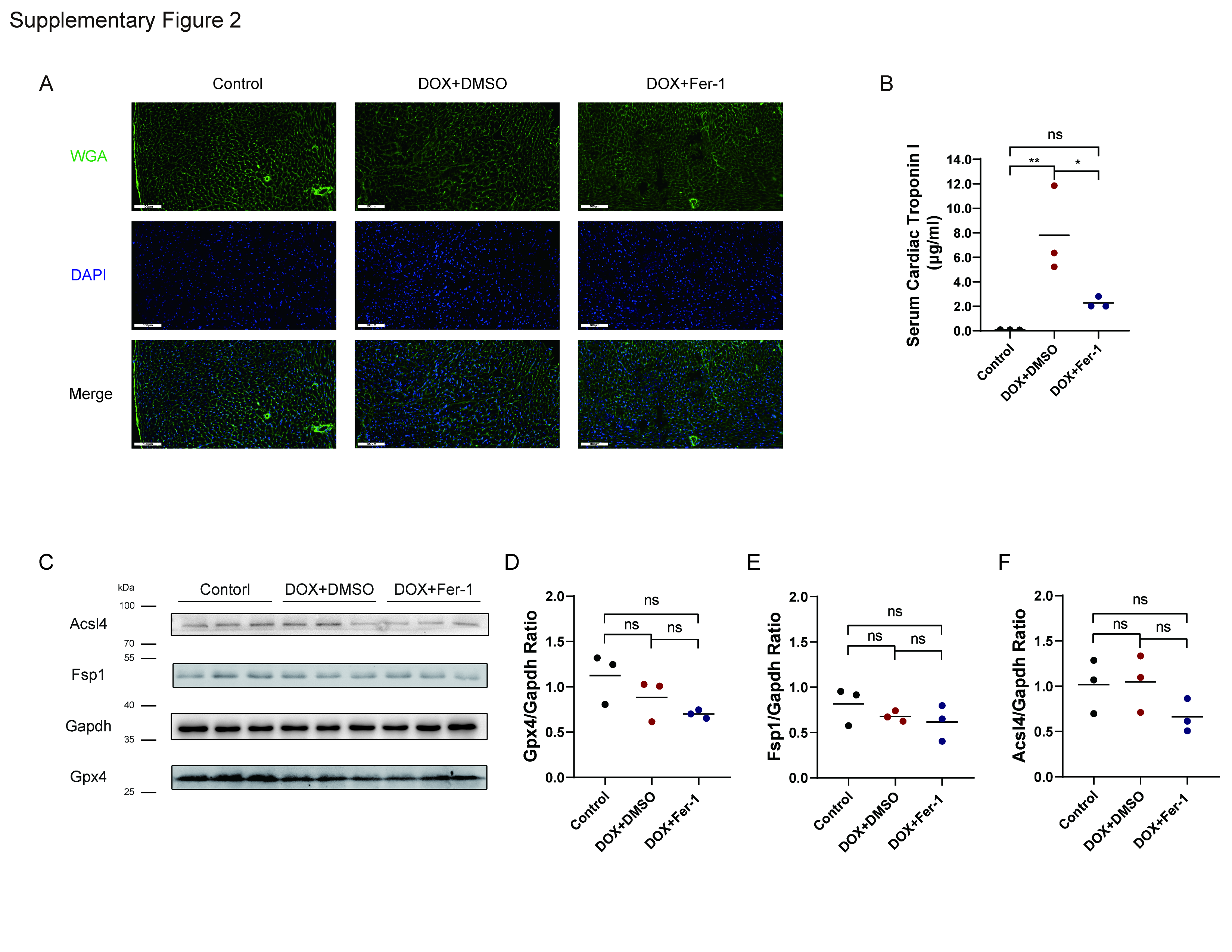

Supplement: Supplementary file 3 — Supplementary Figure 2 [file 41419_2020_2948_MOESM3_ESM.tif]

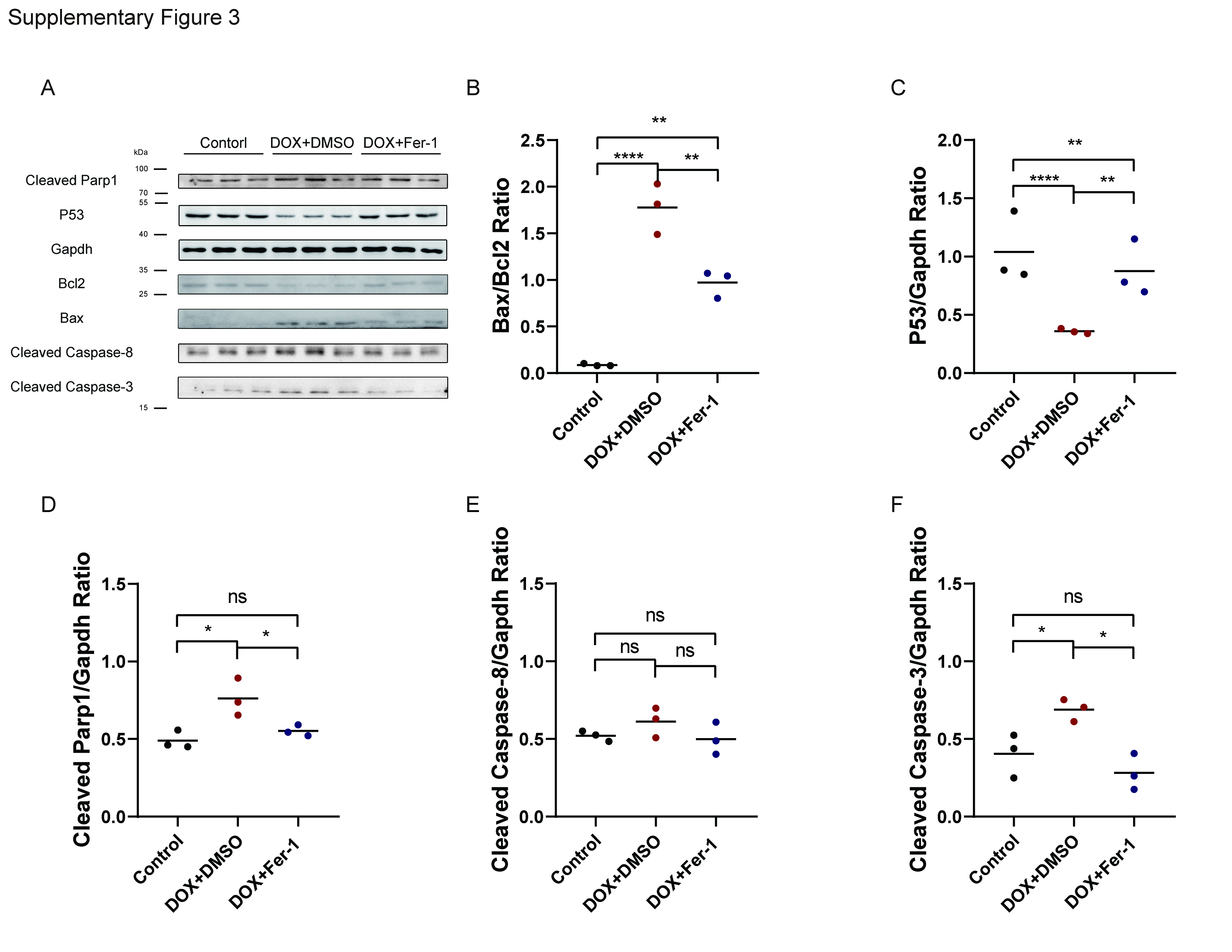

Supplement: Supplementary file 4 — Supplementary Figure 3 [file 41419_2020_2948_MOESM4_ESM.tif]
