# Supplementary material for: Acyl-CoA thioesterase 1 prevents cardiomyocytes from Doxorubicin-induced ferroptosis via shaping the lipid composition
Source: Cell Death Dis. 2020 Sep 15;11(9):756. doi: 10.1038/s41419-020-02948-2 (PMC7492260; doi:10.1038/s41419-020-02948-2)
Supplement: Supplementary file 5 — Supplementary Table 1 [file 41419_2020_2948_MOESM5_ESM.docx]

**Supplementary Table 1.** Expression list of 100 most up regulated genes based on RNA-seq analysis.

| ENTREZID | Gene Symbol | Ensembl Gene ID | Fold Change | P_value |
| --- | --- | --- | --- | --- |
| 140781 | Myh7 | ENSMUSG00000053093 | 32.492 | 3.83E-02 |
| 269643 | Ppp2r2c | ENSMUSG00000029120 | 14.500 | 2.65E-03 |
| 215303 | Camk1g | ENSMUSG00000016179 | 10.250 | 1.88E-02 |
| 50702 | Cfhr1 | ENSMUSG00000057037 | 9.500 | 4.21E-02 |
| 238333 | Samd15 | ENSMUSG00000090812 | 8.800 | 3.72E-02 |
| 30925 | Slamf6 | ENSMUSG00000015314 | 8.500 | 1.69E-02 |
| 53624 | Cldn7 | ENSMUSG00000018569 | 8.250 | 3.84E-02 |
| 100342 | Tent5b | ENSMUSG00000046694 | 8.000 | 3.03E-04 |
| 17858 | Mx2 | ENSMUSG00000023341 | 7.900 | 4.69E-02 |
| 68891 | Cd177 | ENSMUSG00000052212 | 7.500 | 3.92E-02 |
| 19716 | Bex1 | ENSMUSG00000050071 | 7.336 | 1.08E-05 |
| 229599 | Ciart | ENSMUSG00000038550 | 7.288 | 4.66E-03 |
| 192136 | Sugct | ENSMUSG00000055137 | 7.125 | 1.56E-02 |
| 78016 | Ccdc150 | ENSMUSG00000025983 | 6.667 | 3.45E-02 |
| 17063 | Muc13 | ENSMUSG00000022824 | 6.667 | 3.45E-02 |
| 246727 | Oas3 | ENSMUSG00000032661 | 6.643 | 3.59E-02 |
| 14916 | Guca2b | ENSMUSG00000032978 | 6.500 | 3.63E-02 |
| 404337 | Olfr1383 | ENSMUSG00000107417 | 6.208 | 3.08E-02 |
| 217217 | Asb16 | ENSMUSG00000034768 | 6.125 | 1.85E-03 |
| 18741 | Pitx2 | ENSMUSG00000028023 | 6.000 | 2.01E-02 |
| 381853 | Gipr | ENSMUSG00000030406 | 5.692 | 2.50E-02 |
| 632671 | Vmn2r18 | ENSMUSG00000091794 | 5.500 | 4.87E-02 |
| 103149 | Upb1 | ENSMUSG00000033427 | 5.478 | 1.71E-02 |
| 14715 | Gnrhr | ENSMUSG00000029255 | 5.385 | 4.12E-02 |
| 252967 | Ropn1l | ENSMUSG00000022236 | 5.333 | 3.23E-02 |
| 15209 | Hesx1 | ENSMUSG00000040726 | 5.273 | 3.07E-02 |
| 14432 | Gap43 | ENSMUSG00000047261 | 5.250 | 1.77E-03 |
| 231507 | Plac8 | ENSMUSG00000029322 | 5.193 | 4.79E-02 |
| 75304 | 4930563E22Rik | NA | 5.000 | 2.94E-02 |
| 13170 | Dbp | ENSMUSG00000059824 | 4.957 | 3.96E-03 |
| 102639543 | Ifi206 | NA | 4.925 | 3.59E-02 |
| 26886 | Cenph | ENSMUSG00000045273 | 4.889 | 4.67E-02 |
| 73472 | Spata18 | ENSMUSG00000029155 | 4.636 | 3.52E-02 |
| 20293 | Ccl12 | ENSMUSG00000035352 | 4.620 | 4.15E-03 |
| 102635655 | Gm28375 | NA | 4.438 | 7.42E-03 |
| 100417675 | Nlrp5-ps | ENSMUSG00000041596 | 4.435 | 1.25E-03 |
| 28250 | Slco1a4 | ENSMUSG00000030237 | 4.400 | 2.62E-02 |
| 100637 | N4bp2l1 | ENSMUSG00000041132 | 4.329 | 2.85E-02 |
| 69350 | 1700003G18Rik | ENSMUSG00000087621 | 4.281 | 3.36E-02 |
| 23886 | Gdf15 | ENSMUSG00000038508 | 4.250 | 1.38E-02 |
| 108167415 | Gm45978 | NA | 4.154 | 7.54E-03 |
| 626359 | Wdr93 | ENSMUSG00000039099 | 4.083 | 1.60E-02 |
| 258783 | Olfr920 | ENSMUSG00000061039 | 4.067 | 3.32E-02 |
| 234421 | Cib3 | ENSMUSG00000074240 | 4.000 | 5.46E-03 |
| 71724 | Aox3 | ENSMUSG00000064294 | 4.000 | 1.81E-03 |
| 230899 | Nppa | ENSMUSG00000041616 | 3.985 | 2.82E-02 |
| 20871 | Aurkc | ENSMUSG00000070837 | 3.950 | 9.83E-03 |
| 22147 | Tuba3b | ENSMUSG00000067338 | 3.900 | 3.98E-02 |
| 320719 | 6030443J06Rik | ENSMUSG00000097207 | 3.881 | 1.81E-02 |
| 226970 | Arhgef4 | ENSMUSG00000037509 | 3.833 | 1.23E-02 |
| 13390 | Dlx1 | ENSMUSG00000041911 | 3.800 | 2.58E-02 |
| 27047 | Omd | ENSMUSG00000048368 | 3.750 | 3.21E-03 |
| 14939 | Gzmb | ENSMUSG00000015437 | 3.742 | 1.92E-02 |
| 19193 | Pipox | ENSMUSG00000017453 | 3.667 | 2.25E-02 |
| 18407 | Orm3 | ENSMUSG00000028359 | 3.643 | 4.76E-02 |
| 69513 | 1700030C10Rik | ENSMUSG00000099759 | 3.586 | 1.07E-02 |
| 64380 | Ms4a4c | ENSMUSG00000024675 | 3.564 | 4.91E-02 |
| 21743 | Inmt | ENSMUSG00000003477 | 3.534 | 3.48E-03 |
| 269788 | Lhfpl4 | ENSMUSG00000042873 | 3.429 | 3.70E-02 |
| 237553 | Trhde | ENSMUSG00000050663 | 3.400 | 2.72E-02 |
| 69479 | 1700029J07Rik | ENSMUSG00000071103 | 3.362 | 2.28E-03 |
| 209334 | Gen1 | ENSMUSG00000051235 | 3.323 | 9.39E-03 |
| 245450 | Slitrk2 | ENSMUSG00000036790 | 3.300 | 4.79E-02 |
| 100040599 | Gm15319 | ENSMUSG00000074449 | 3.240 | 1.63E-02 |
| 18768 | Pkib | ENSMUSG00000019876 | 3.179 | 2.66E-03 |
| 20310 | Cxcl2 | ENSMUSG00000058427 | 3.175 | 4.40E-02 |
| 106763 | Ttbk1 | ENSMUSG00000015599 | 3.154 | 1.37E-02 |
| 50500 | Ttpa | ENSMUSG00000073988 | 3.132 | 1.58E-02 |
| 433698 | Fam205a1 | ENSMUSG00000078721 | 3.077 | 5.35E-03 |
| 11624 | Ahrr | ENSMUSG00000021575 | 3.074 | 1.40E-05 |
| 14695 | Gnb3 | ENSMUSG00000023439 | 3.063 | 1.73E-02 |
| 107765 | Ankrd1 | ENSMUSG00000024803 | 3.029 | 3.57E-05 |
| 18158 | Nppb | ENSMUSG00000029019 | 3.019 | 1.07E-02 |
| 73453 | 1700067K01Rik | ENSMUSG00000046408 | 2.931 | 3.35E-02 |
| 16618 | Klk1b26 | ENSMUSG00000053719 | 2.895 | 3.35E-02 |
| 18260 | Ocln | ENSMUSG00000021638 | 2.882 | 8.02E-03 |
| 69297 | Lrrc46 | ENSMUSG00000020878 | 2.879 | 4.52E-02 |
| 623121 | Ifi213 | ENSMUSG00000073491 | 2.857 | 4.72E-02 |
| 75196 | Ankrd7 | ENSMUSG00000029517 | 2.842 | 1.48E-02 |
| 14711 | Gnmt | ENSMUSG00000002769 | 2.838 | 6.93E-03 |
| 18627 | Per2 | ENSMUSG00000055866 | 2.818 | 1.78E-03 |
| 73747 | Shld1 | ENSMUSG00000044991 | 2.739 | 1.59E-03 |
| 19275 | Ptprn | ENSMUSG00000026204 | 2.730 | 1.71E-02 |
| 68846 | Rnf208 | ENSMUSG00000044628 | 2.729 | 3.31E-02 |
| 14049 | Eya2 | ENSMUSG00000017897 | 2.706 | 1.31E-03 |
| 74041 | Ddias | ENSMUSG00000030641 | 2.690 | 2.90E-02 |
| 242505 | Rasef | ENSMUSG00000043003 | 2.674 | 1.20E-03 |
| 67492 | Zfand4 | ENSMUSG00000108273 | 2.671 | 3.38E-03 |
| 100040972 | Tceal7 | ENSMUSG00000079428 | 2.648 | 2.69E-02 |
| 18628 | Per3 | ENSMUSG00000028957 | 2.640 | 1.78E-05 |
| 102635549 | Gm32853 | NA | 2.616 | 2.33E-02 |
| 17961 | Nat2 | ENSMUSG00000051147 | 2.597 | 3.86E-02 |
| 432769 | Zfp708 | ENSMUSG00000100235 | 2.542 | 7.02E-03 |
| 16625 | Serpina3c | ENSMUSG00000066361 | 2.529 | 2.99E-04 |
| 15958 | Ifit2 | ENSMUSG00000045932 | 2.526 | 3.02E-02 |
| 16156 | Il11 | ENSMUSG00000004371 | 2.516 | 4.78E-02 |
| 76223 | Agbl3 | ENSMUSG00000038836 | 2.516 | 6.30E-03 |
| 74574 | Lvrn | ENSMUSG00000024481 | 2.513 | 2.40E-02 |
| 73610 | Zfp433 | ENSMUSG00000096795 | 2.505 | 3.61E-02 |
| 100041874 | Gm3558 | ENSMUSG00000079364 | 2.500 | 4.02E-02 |
