# Supplementary material for: Acyl-CoA thioesterase 1 prevents cardiomyocytes from Doxorubicin-induced ferroptosis via shaping the lipid composition
Source: Cell Death Dis. 2020 Sep 15;11(9):756. doi: 10.1038/s41419-020-02948-2 (PMC7492260; doi:10.1038/s41419-020-02948-2)
Supplement: Supplementary file 6 — Supplementary Table 2 [file 41419_2020_2948_MOESM6_ESM.docx]

**Supplementary Table 2.** Expression list of 100 most down regulated genes based on RNA-seq analysis.

| ENTREZID | Gene Symbol | Ensembl Gene ID | Fold Change | P_value |
| --- | --- | --- | --- | --- |
| 381284 | Crocc2 | ENSMUSG00000084989 | 0.036 | 2.45E-03 |
| 15413 | Hoxb5 | ENSMUSG00000038700 | 0.074 | 1.38E-02 |
| 15165 | Hcn1 | ENSMUSG00000021730 | 0.081 | 1.25E-03 |
| 241128 | Fam124b | ENSMUSG00000043230 | 0.093 | 1.40E-03 |
| 14525 | Gcsam | ENSMUSG00000022659 | 0.111 | 1.51E-02 |
| 105245981 | Gm41349 | NA | 0.111 | 1.49E-02 |
| 15221 | Foxd3 | ENSMUSG00000067261 | 0.117 | 6.71E-03 |
| 105245580 | A930018O16Rik | NA | 0.117 | 6.20E-03 |
| 626834 | Klk13 | ENSMUSG00000054046 | 0.120 | 1.52E-02 |
| 80893 | Tmprss5 | ENSMUSG00000032268 | 0.122 | 4.86E-02 |
| 71920 | Epgn | ENSMUSG00000035020 | 0.125 | 4.96E-03 |
| 70163 | Lypd8 | ENSMUSG00000013643 | 0.131 | 9.02E-03 |
| 240913 | Adamts4 | ENSMUSG00000006403 | 0.132 | 6.48E-03 |
| 51811 | Clec4f | ENSMUSG00000014542 | 0.133 | 4.40E-02 |
| 13011 | Cst7 | ENSMUSG00000068129 | 0.133 | 4.22E-03 |
| 100041262 | Gm3239 | NA | 0.142 | 1.61E-02 |
| 237868 | Sarm1 | ENSMUSG00000050132 | 0.143 | 6.47E-03 |
| 66311 | Cenpw | ENSMUSG00000075266 | 0.151 | 1.84E-02 |
| 26970 | Pla2g2e | ENSMUSG00000028751 | 0.153 | 2.62E-02 |
| 353237 | Pcdhac2 | ENSMUSG00000102697 | 0.157 | 3.07E-02 |
| 93892 | Pcdhb21 | ENSMUSG00000044022 | 0.163 | 2.48E-02 |
| 108956 | Apol7c | ENSMUSG00000044309 | 0.172 | 4.79E-02 |
| 15401 | Hoxa4 | ENSMUSG00000000942 | 0.177 | 2.03E-02 |
| 18430 | Oxtr | ENSMUSG00000049112 | 0.188 | 9.59E-03 |
| 545652 | Gm13275 | ENSMUSG00000099518 | 0.190 | 9.03E-04 |
| 20974 | Syngr3 | ENSMUSG00000007021 | 0.192 | 4.34E-02 |
| 108168740 | Gm46731 | NA | 0.208 | 2.19E-02 |
| 319171 | Hist1h2ap | ENSMUSG00000094777 | 0.222 | 2.24E-02 |
| 74438 | Clvs1 | ENSMUSG00000041216 | 0.228 | 5.30E-03 |
| 14462 | Gata3 | ENSMUSG00000015619 | 0.243 | 3.10E-02 |
| 108167883 | Gm46270 | NA | 0.245 | 8.03E-03 |
| 110902 | Chrna2 | ENSMUSG00000022041 | 0.249 | 5.07E-04 |
| 22402 | Wisp1 | ENSMUSG00000005124 | 0.253 | 4.45E-03 |
| 381680 | Nxpe5 | ENSMUSG00000047592 | 0.253 | 9.25E-03 |
| 381417 | Gm14085 | ENSMUSG00000079071 | 0.256 | 2.07E-02 |
| 233529 | Kctd14 | ENSMUSG00000051727 | 0.256 | 2.71E-02 |
| 625591 | Cldn34c2 | ENSMUSG00000095474 | 0.258 | 2.17E-02 |
| 17306 | Sypl2 | ENSMUSG00000027887 | 0.262 | 1.03E-03 |
| 78354 | 2210407C18Rik | ENSMUSG00000037145 | 0.273 | 1.71E-02 |
| 329065 | Scd4 | ENSMUSG00000050195 | 0.274 | 4.08E-05 |
| 230779 | Serinc2 | ENSMUSG00000023232 | 0.275 | 5.34E-03 |
| 232790 | Oscar | ENSMUSG00000054594 | 0.280 | 4.10E-02 |
| 54141 | Spag5 | ENSMUSG00000002055 | 0.286 | 2.69E-02 |
| 317750 | Slc24a5 | ENSMUSG00000035183 | 0.287 | 4.85E-02 |
| 15400 | Hoxa3 | ENSMUSG00000079560 | 0.290 | 2.52E-02 |
| 194309 | Vps37d | ENSMUSG00000043614 | 0.293 | 1.51E-02 |
| 54612 | Sfrp5 | ENSMUSG00000018822 | 0.296 | 2.67E-02 |
| 11865 | Arntl | ENSMUSG00000055116 | 0.304 | 1.44E-03 |
| 16499 | Kcnab3 | ENSMUSG00000018470 | 0.304 | 9.65E-03 |
| 12478 | Cd19 | ENSMUSG00000030724 | 0.304 | 2.08E-02 |
| 12984 | Csf2rb2 | ENSMUSG00000071714 | 0.304 | 3.21E-03 |
| 22092 | Rsph1 | ENSMUSG00000024033 | 0.304 | 4.74E-03 |
| 213945 | Col28a1 | ENSMUSG00000068794 | 0.305 | 2.42E-04 |
| 13076 | Cyp1a1 | ENSMUSG00000032315 | 0.307 | 2.00E-02 |
| 229474 | Fhdc1 | ENSMUSG00000041842 | 0.310 | 1.73E-02 |
| 12960 | Crybb1 | ENSMUSG00000029343 | 0.314 | 6.85E-04 |
| 545554 | Ankrd34a | ENSMUSG00000049097 | 0.318 | 4.00E-03 |
| 19378 | Aldh1a2 | ENSMUSG00000013584 | 0.320 | 3.66E-03 |
| 213696 | Duoxa1 | ENSMUSG00000027224 | 0.322 | 3.52E-02 |
| 105247220 | Gm42355 | NA | 0.325 | 2.60E-02 |
| 67749 | Mgarp | ENSMUSG00000037161 | 0.327 | 1.20E-02 |
| 30806 | Adamts8 | ENSMUSG00000031994 | 0.328 | 9.62E-03 |
| 237387 | Lrrc3 | ENSMUSG00000051652 | 0.337 | 1.63E-02 |
| 56696 | Gpr132 | ENSMUSG00000021298 | 0.344 | 3.76E-04 |
| 545260 | Arsi | ENSMUSG00000036412 | 0.345 | 1.83E-02 |
| 231842 | Amz1 | ENSMUSG00000050022 | 0.346 | 5.99E-03 |
| 74116 | Pi16 | ENSMUSG00000024011 | 0.346 | 6.61E-03 |
| 20716 | Serpina3n | ENSMUSG00000021091 | 0.347 | 3.50E-03 |
| 101544 | Zfp575 | ENSMUSG00000066721 | 0.350 | 8.59E-03 |
| 57138 | Slc12a5 | ENSMUSG00000017740 | 0.351 | 4.72E-03 |
| 15412 | Hoxb4 | ENSMUSG00000038692 | 0.355 | 2.25E-02 |
| 30936 | Slc46a2 | ENSMUSG00000028386 | 0.356 | 5.26E-03 |
| 73710 | Tubb2b | ENSMUSG00000045136 | 0.359 | 2.46E-02 |
| 118454 | Gjc2 | ENSMUSG00000043448 | 0.360 | 4.26E-02 |
| 22061 | Trp63 | ENSMUSG00000022510 | 0.361 | 1.80E-02 |
| 85030 | Tnfrsf25 | ENSMUSG00000024793 | 0.361 | 2.41E-02 |
| 171504 | Apobr | ENSMUSG00000042759 | 0.363 | 4.12E-04 |
| 93716 | Pcdhga8 | ENSMUSG00000103897 | 0.364 | 4.41E-02 |
| 14961 | H2-Ab1 | ENSMUSG00000073421 | 0.364 | 1.43E-03 |
| 78416 | Rnase6 | ENSMUSG00000021880 | 0.368 | 2.15E-02 |
| 100689 | Spon2 | ENSMUSG00000037379 | 0.368 | 1.63E-02 |
| 71733 | Susd2 | ENSMUSG00000006342 | 0.368 | 2.60E-02 |
| 214058 | Megf11 | ENSMUSG00000036466 | 0.368 | 2.83E-02 |
| 14969 | H2-Eb1 | ENSMUSG00000060586 | 0.370 | 3.58E-02 |
| 12062 | Bdkrb2 | ENSMUSG00000021070 | 0.375 | 2.23E-02 |
| 17381 | Mmp12 | ENSMUSG00000049723 | 0.377 | 2.58E-02 |
| 26897 | Acot1 | ENSMUSG00000072949 | 0.379 | 8.39E-04 |
| 76293 | Mfap4 | ENSMUSG00000042436 | 0.379 | 1.91E-02 |
| 380694 | Ccnjl | ENSMUSG00000044707 | 0.382 | 8.74E-06 |
| 18030 | Nfil3 | ENSMUSG00000056749 | 0.383 | 1.08E-03 |
| 16069 | Jchain | ENSMUSG00000067149 | 0.385 | 6.72E-03 |
| 18082 | Nipsnap1 | ENSMUSG00000034285 | 0.386 | 4.01E-02 |
| 12516 | Cd7 | ENSMUSG00000025163 | 0.387 | 3.94E-02 |
| 68026 | Pclaf | ENSMUSG00000040204 | 0.387 | 1.56E-03 |
| 19713 | Ret | ENSMUSG00000030110 | 0.389 | 7.52E-05 |
| 14065 | F2rl3 | ENSMUSG00000050147 | 0.390 | 3.21E-02 |
| 66650 | Nepn | ENSMUSG00000038624 | 0.390 | 1.17E-02 |
| 230145 | Galnt12 | ENSMUSG00000039774 | 0.390 | 4.84E-02 |
| 545030 | Wdfy4 | ENSMUSG00000051506 | 0.393 | 1.29E-02 |
| 192897 | Itgb4 | ENSMUSG00000020758 | 0.395 | 1.01E-02 |
