# Supplementary material for: Acyl-CoA thioesterase 1 prevents cardiomyocytes from Doxorubicin-induced ferroptosis via shaping the lipid composition
Source: Cell Death Dis. 2020 Sep 15;11(9):756. doi: 10.1038/s41419-020-02948-2 (PMC7492260; doi:10.1038/s41419-020-02948-2)
Supplement: Supplementary file 7 — Supplementary Table 3 [file 41419_2020_2948_MOESM7_ESM.docx]

**Supplementary Table 3**. Expression list of Ferroptosis-related genes based on RNA-seq analysis.

| Gene Information | | | Control | | | DOX | | |
| --- | --- | --- | --- | --- | --- | --- | --- | --- |
| ENTREZID | Gene Symbol | Ensembl Gene ID | Con1 | Con2 | Con3 | Dox1 | Dox2 | Dox3 |
| 71775 | 1300017J02Rik | ENSMUSG00000033688 | 0.06 | 0.06 | 0.06 | 0.06 | 0.05 | 0.34 |
| 14081 | Acsl1 | ENSMUSG00000018796 | 395.21 | 439.86 | 470.08 | 331.34 | 340.01 | 332.9 |
| 74205 | Acsl3 | ENSMUSG00000032883 | 2.1 | 1.82 | 2.57 | 2.24 | 1.82 | 2.55 |
| 50790 | Acsl4 | ENSMUSG00000031278 | 3.28 | 2.92 | 3.41 | 2.82 | 3.43 | 4.05 |
| 433256 | Acsl5 | ENSMUSG00000024981 | 8.25 | 8.11 | 8.28 | 7.31 | 8.09 | 10.16 |
| 216739 | Acsl6 | ENSMUSG00000020333 | 3.18 | 4.44 | 4.63 | 3.86 | 4.41 | 4.54 |
| 11687 | Alox15 | ENSMUSG00000018924 | NA | NA | NA | NA | NA | NA |
| 11793 | Atg5 | ENSMUSG00000038160 | 14.41 | 11.32 | 13.63 | 10.41 | 11.26 | 12.93 |
| 74244 | Atg7 | ENSMUSG00000030314 | 5.19 | 6.4 | 6.3 | 7.37 | 6.45 | 6.35 |
| 12870 | Cp | ENSMUSG00000003617 | 19.88 | 27.59 | 33.65 | 43.4 | 29.42 | 35.64 |
| 13058 | Cybb | ENSMUSG00000015340 | 3.54 | 3.94 | 3.82 | 2.46 | 2.76 | 3.81 |
| 14319 | Fth1 | ENSMUSG00000024661 | 2556.56 | 2477.61 | 2275 | 2965.69 | 2558.02 | 2264.09 |
| 14325 | Ftl1 | ENSMUSG00000050708 | 418.04 | 417.26 | 329.25 | 321.75 | 332.83 | 428.69 |
| 67634 | Ftmt | ENSMUSG00000024510 | NA | NA | NA | NA | NA | NA |
| 14629 | Gclc | ENSMUSG00000032350 | 4.93 | 4.2 | 4.76 | 6.7 | 6.33 | 7.5 |
| 14630 | Gclm | ENSMUSG00000028124 | 10.19 | 12.2 | 11.22 | 12.43 | 9.65 | 10.31 |
| 625249 | Gpx4 | ENSMUSG00000075706 | 979.03 | 907.86 | 799.46 | 889.8 | 867.49 | 750.34 |
| 14854 | Gss | ENSMUSG00000027610 | 7.89 | 9.73 | 7.82 | 9.63 | 8.26 | 10.43 |
| 15368 | Hmox1 | ENSMUSG00000005413 | 11.88 | 9.19 | 8.63 | 5.55 | 7.09 | 8.69 |
| 1.01E+08 | LOC100862446 | NA | NA | NA | NA | NA | NA | NA |
| 14792 | Lpcat3 | ENSMUSG00000004270 | 34.57 | 32 | 31.92 | 24.86 | 24.23 | 22.13 |
| 66734 | Map1lc3a | ENSMUSG00000027602 | 585.89 | 544.7 | 495.39 | 536.58 | 544.95 | 477.3 |
| 67443 | Map1lc3b | ENSMUSG00000031812 | 192.39 | 183.15 | 169.04 | 189.79 | 181.12 | 178.39 |
| Gene Information | | | Control | | | DOX | | |
| ENTREZID | Gene.Symbol | Ensembl.Gene.ID | Con1 | Con2 | Con3 | Dox1 | Dox2 | Dox3 |
| 27057 | Ncoa4 | ENSMUSG00000056234 | 41.22 | 38.84 | 37.58 | 40.75 | 41.97 | 37.09 |
| 23983 | Pcbp1 | ENSMUSG00000051695 | 106.77 | 110.27 | 104.11 | 104.44 | 103.15 | 82.52 |
| 18521 | Pcbp2 | ENSMUSG00000056851 | 248.47 | 232.06 | 228.23 | 253.97 | 237.73 | 224.81 |
| 19122 | Prnp | ENSMUSG00000079037 | 83.03 | 85.92 | 82.62 | 82.37 | 88.31 | 80.66 |
| 20229 | Sat1 | ENSMUSG00000025283 | 20.89 | 21.08 | 16.48 | 16.41 | 20.61 | 24.93 |
| 69215 | Sat2 | ENSMUSG00000069835 | 3.01 | 2.76 | 3.33 | 4.18 | 3.86 | 3.01 |
| 18174 | Slc11a2 | ENSMUSG00000023030 | 12.06 | 12.19 | 11.6 | 10.28 | 11.81 | 9.71 |
| 213053 | Slc39a14 | ENSMUSG00000022094 | 21.88 | 23.06 | 25.65 | 21.12 | 17.68 | 15.78 |
| 67547 | Slc39a8 | ENSMUSG00000053897 | 0.46 | 0.72 | 0.45 | 0.48 | 0.43 | 0.84 |
| 17254 | Slc3a2 | ENSMUSG00000010095 | 21.39 | 23.66 | 20.87 | 21.31 | 22.3 | 18.94 |
| 53945 | Slc40a1 | ENSMUSG00000025993 | 8.76 | 9.37 | 8.75 | 7.66 | 6.55 | 7.77 |
| 26570 | Slc7a11 | ENSMUSG00000027737 | NA | NA | NA | NA | NA | NA |
| 68428 | Steap3 | ENSMUSG00000026389 | 28.98 | 29.47 | 25.74 | 17.73 | 22.75 | 19.39 |
| 22042 | Tfrc | ENSMUSG00000022797 | 25.19 | 20.86 | 29.93 | 13.06 | 20.68 | 16.6 |
| 22041 | Trf | ENSMUSG00000032554 | 25.57 | 28.45 | 19.57 | 14.65 | 17.83 | 33.47 |
| 22059 | Trp53 | ENSMUSG00000059552 | 10.46 | 11 | 11.1 | 11.32 | 11.94 | 12.05 |
| 22334 | Vdac2 | ENSMUSG00000021771 | 369.65 | 333.13 | 334.99 | 396.74 | 383.13 | 384.52 |
| 22335 | Vdac3 | ENSMUSG00000008892 | 360.56 | 280.49 | 299.02 | 338.16 | 339.72 | 334.47 |
