# Supplementary material for: Acyl-CoA thioesterase 1 prevents cardiomyocytes from Doxorubicin-induced ferroptosis via shaping the lipid composition
Source: Cell Death Dis. 2020 Sep 15;11(9):756. doi: 10.1038/s41419-020-02948-2 (PMC7492260; doi:10.1038/s41419-020-02948-2)
Supplement: Supplementary file 8 — Supplementary Table 4 [file 41419_2020_2948_MOESM8_ESM.docx]

**Supplementary Table 4**. Anti-bodies Information.

| Anti-bodies | Vendor | Catalog NO. |
| --- | --- | --- |
| Acot1 | Abcam | ab100915 |
| P53 | Abcam | ab179477 |
| Gapdh | Abcam | ab9485 |
| Cleaved Parp1 | CST | 5625 |
| Cleaved Caspase-8 | CST | 8592 |
| Cleaved Caspase-3 | CST | 9664 |
| Bcl2 | Proteintech | [26593-1-AP](http://www.ptgcn.com/products/Bcl2-Antibody-26593-1-AP.htm) |
| Bax | Proteintech | [50599-2-Ig](http://www.ptgcn.com/products/BAX-Antibody-50599-2-Ig.htm) |
| Fsp1 | Proteintech | 20886-1-AP |
| Gpx4 | Proteintech | 14432-1-AP |
| Acsl4 | SantaCruz | sc-365230 |
